# Supplementary material for: Cross-sectional Survey of Medical student Attitudes to Research and Training pathways (SMART) in the UK: study protocol
Source: BMJ Open. 2021 Sep 2;11(9):e050104. doi: 10.1136/bmjopen-2021-050104 (PMC8413964; doi:10.1136/bmjopen-2021-050104)
Supplement: Supplementary data [file bmjopen-2021-050104supp001.pdf]

Appendix S1

Proforma of questionnaire, to be converted to digital format for administration. Text in red describes the available answers for each question.

Q1a. Which medical school do you attend? (If currently intercalating at a separate university, please give the university from which you will receive your main degree).

☐ Please select your university from the dropdown list *as in appendix S2*

Q1b. Which year of medical school are you currently in? (Ordinal)

☐ Please select which year of your medical degree you are currently enrolled in (First/Second/Third/ /Year 4 of 5/ Year 4 of 6/Year 5 of 6/ Year 5 of 5 /Year 6 of 6/ Intercalation/ Graduate Entry first/ Graduate Entry second/ Graduate Entry third/ Graduate Entry fourth

Q2. Have you already completed an academic degree (Bachelor's/Masters/Doctorate)? (binary)

☐ Please select whether you currently already have a completed higher education degree, such as a Bachelor's, Master's, or Doctorate. (YES/NO)

Q3. If you answered yes to Question 2, please select all those degrees you currently have. (Nominal/Ordinal)

☐ Please select all degrees you currently have. (Bachelor's – in a scientific degree/Bachelor's – in an arts degree/Master's – in a scientific degree/Master's – in an arts degree/Doctorate/ Other (please type))

Q4. Choose one option that best describes your ethnic group or background? (Nominal)

☐ Please select which ethnic group you most strongly identify as. (English, Welsh, Scottish, Northern Irish, British/Irish/Gypsy or Irish Traveller/Any other White background/ White and Black Carribean/ White and Black African/ White and Asian/ Any other Mixed or Multiple ethnic background/ Indian/ Pakistani/ Bangladeshi/ Chinese/ Any other Asian background/ African/Caribbean/ Any other Black, African, or Caribbean background/Arab/ Any other ethnic group)

*Note, list taken from government list of ethnic groups https://www.ethnicity-facts-figures.service.gov.uk/style-guide/ethnic-groups

Q5. What best describes your gender (Nominal)

☐ Female/ Male/ Prefer not to say/ Prefer to self-describe (free text)

Q6. Do you identify as LGBTQ+? (Binary)

☐ Please select whether you identify as LGBTQ+ (Lesbian, Gay, Bisexual, Transgender, Queer/Questioning, or any other part of the LGBTQ+ community). (YES/NO)

Q7. During school, at any point, were you eligible for free school meals? (binary)

☐ Please select whether you were, at any point, eligible for free school meals during school. (Yes/No)

Q8. How many of your first degree relatives are or have been a healthcare professional*? (Ratio/Ordinal)

☐ Please select the number of relatives who currently or have ever identify/identified as healthcare professionals. (0,1,2,3,4,5,6,7,8,9,10,10+)

*Healthcare professional defined according to WHO international classification of healthcare workers as professionals who study, advise on or provide preventive, curative, rehabilitative and promotional health services based on an extensive body of theoretical and factual knowledge in diagnosis and treatment of disease and other health problems. This includes:

- General medical practitioners
- Specialist medical practitioners
- Nursing professionals
- Midwifery professionals
- Traditional and complementary medicine professionals
- Paramedical practitioners
- Dentists
- Pharmacists
- Environmental and occupational health and hygiene professionals
- Physiotherapists

- Dieticians and nutritionists
- Optometrists and ophthalmic opticians

Q8b. If you answered >0 Question 8, how many of your relatives are or have been held an academic position* in the healthcare environment? (Ratio/ordinal)

☐ Please select all the number of relatives who currently or have ever identify/identified as academics in the healthcare environment. (0,1,2,3,4,5,6,7,8,9,10,10+)

*Academic position defined as working within a research or teaching capacity within a higher education institute. This includes, but is not limited, to, professors, readers, senior lecturers, lecturers, post-doctoral researchers, research fellows, teaching fellows

Q9. How many of your first degree relatives are or have been in academia? (Ratio/ordinal)

☐ Please select the number of relatives who currently or have ever identify/identified as being in academia. (0,1,2,3,4,5,6,7,8,9,10,10+)

Q9b. If you answered >0 Question 9, how many of your relatives are or have been held an academic position in the healthcare environment? (Ratio/ordinal)

☐ Please select all the number of relatives who currently or have ever identify/identified as academics in the healthcare environment. (0,1,2,3,4,5,6,7,8,9,10,10+)

*Academic position defined as working within a research or teaching capacity within a higher education institute. This includes, but is not limited, to, professors, readers, senior lecturers, lecturers, post-doctoral researchers, research fellows, teaching fellows

Q11. In which area did you undertake the majority of your pre-university education?

☐ Please select the geographical area where you undertook the majority of your education before university (UK, EU, outside EU)

Q12. How well do you feel your medical school has educated you about research? (Ordinal)

☐ Please select which best represents your opinion. (Not well at all/ Less than adequately/Adequately/More than adequately/Very well)

Q13. How much research have you undertaken to date? (Ordinal)

☐ Please select how much research you feel you have undertaken to date. (None/A little bit/ A fair amount/Quite a lot/A very significant body of research)

Q14. If you have undertaken any research, was any of this a compulsory part of your degree? (Binary)

☐ Please select whether any research you have done included a compulsory part of your degree. (Yes/No)

Q15. If you have not yet been involved in voluntary research, what have been the barriers preventing this? (Nominal)

☐ Please describe the barriers that have prevented you from undertaking research outside of any compulsory degree obligation. (Free text up to 500 words)

Q16. Please tick any of the following that apply to your research. You may tick more than one box or none at all : (Nominal)

☐ Please tick any of the following that apply to your research: (QIP/audit/basic science project/ clinical project/ co-author on original paper in peer reviewed journal/ co-author on any publications related to research/ named collaborator on original paper in peer reviewed journal/ named collaborator on any publication related to research/ presented a poster/ given an oral presentation/other (please type))

Q17. Why did/do you do research? (Nominal)

☐ Please tick any of the following that apply to your research:. (Interest in scientific problems/Interest in the subject/Personal development/Extra income/contribution to better health care/Improving critical thinking/Career progression/Intellectual stimulation/Feel obliged to do it/Other (please type)

Q18. How much do you think research is useful in combination with your medical studies? (Ordinal)

☐ Please select to what degree you feel research is useful in combination with your medical studies. (Not at all useful/ Somewhat useful/ A little useful/A lot useful/Very useful)

Q19. How difficult is it to combine research with your medical studies? (Ordinal)

☐ Please select how difficult you find it to integrate research with your medical studies. (Not at all difficult/Somewhat difficult/A little difficult/ A lot difficult/ Very difficult)

Q20. How much do you agree with the statement: "I wish to pursue an academic career ." (Ordinal)

☐ Please select how strongly you intend to pursue an academic career. (Strongly disagree/ Somewhat disagree/No opinion/Somewhat agree/Strongly agree)

Q21. How much do you agree with the statement: "I wish to pursue an academic training pathway ." (Ordinal)

Please select how strongly you intend to pursue an academic career. (Strongly disagree/ Somewhat disagree/No opinion/Somewhat agree/Strongly agree)

Q22. How much do you agree with the statement: "I would be interested in undertaking (more) research in the future." (Ordinal)

☐ Please select how strongly you agree with the above statement. (Strongly disagree/ Somewhat disagree/No opinion/Somewhat agree/Strongly agree)

Q23. What would encourage your involvement in research in the future? (Ordinal)

☐ Please tick all that apply
(More time/More incentives/ Easier access to research groups and projects/ Clearer information about how to get involved/ Clearer information about benefits of research/ Guaranteed rewards e.g. publication or presentation/ Other (please type)

<u>Appendix S2</u>

A list of medical schools recognised by the GMC as of 1st January 2020:
The University of Aberdeen
The University of Birmingham
The University of Bristol
The University of Buckingham
The University of Cambridge
Cardiff University
The University of Dundee
The University of East Anglia
The University of Edinburgh
University of Exeter Medical School
The University of Glasgow
The Imperial College of Science, Technology and Medicine
Keele University
King’s College London
Lancaster University
The University of Leeds
The University of Leicester
The University of Liverpool
The University of London
The University of Manchester
The University of Newcastle
The University of Nottingham
The University of Oxford
Plymouth University Peninsula Schools of Medicine and Dentistry
Queen Mary University of London
The Queen’s University of Belfast
St George's Hospital Medical School
Swansea University
The University of Sheffield
The University of Southampton
University College London
The University of Warwick
A combination of the University of Brighton and the University of Sussex
A combination of the University of Hull and the University of York

Appendix S3

This document outlines the participant facing information used in recruitment for the SMART study

1. **Enrolment and 1st Survey Phase:** Link shared via email and social media
    a. Initiation: 1st – 14th November
2. **1st and 2nd Follow up for 1st Survey**
    a. 14th November – 30th November
3. **3rd Follow up for 1st Survey**
    a. 31st November – 1st January

1. Initial email to students

Dear Students,

We are a group of researchers from across the country who are interested in understanding the medical student perceptions of research and research-orientated careers.

We invite you to participate in a 15-minute voluntary survey that is collecting information on your views on research and research-orientated careers. When you access the survey, you are going to be asked a variety of questions about your background, your previous experiences of research, and your thoughts on a future research career. Your IP address will not be recorded. All answers will remain anonymous and confidential.

If you wish to partake in our prize draw, please provide your email address when requested by the survey. This email address will not be linked to your answers. More information on the risks and benefits of completing this survey are included in the survey link. **You can access the survey here:**

<INS LINK>

If you have any questions, you can contact a member of the research team by email at soham.bandyopadhyay@st-hildas.ox.ac.uk
Thank you for your consideration.

Many thanks,
<Your name>
SMART team

2. First and Second Follow-up email for 1st survey

Dear Students,

Just a friendly reminder that the SMART survey will remain open until January 1st, 2022. Please take a few moments to participate in this survey if you have not done so yet.

We invite you to participate in a 15-minute voluntary survey that is collecting information on your views on research and research-orientated careers. When you access the survey, you are going to be asked a variety of questions about your background, your previous experiences of research, and your thoughts on a future research career. Your IP address will not be recorded. All answers will remain anonymous and confidential.

If you wish to partake in our prize draw, please provide your email address when requested by the survey. This email address will not be linked to your answers. More information on the risks and benefits of completing this survey are included in the survey link. **You can access the survey here:**

<INS LINK>
If you have any questions, you can contact a member of the research team by email at soham.bandyopadhyay@st-hildas.ox.ac.uk

Thank you for your consideration.

Many thanks,
<Your name>
SMART team

3. Third Follow-up email for 1st survey (sent out the day before the survey closes)

Dear Students,
Just a friendly reminder that the SMART survey will remain open until January 1st, 2022. Please take a few moments to participate in this survey if you have not done so yet.

We invite you to participate in a 15-minute voluntary survey that is collecting information on your views on research and research-orientated careers. When you access the survey, you are going to be asked a variety of questions about your background, your previous experiences of research, and your thoughts on a future research career. Your IP address will not be recorded. All answers will remain anonymous and confidential.

If you wish to partake in our prize draw, please provide your email address when requested by the survey. This email address will not be linked to your answers. More information on the risks and benefits of completing this survey are included in the survey link. **You can access the survey here:**

<INS LINK>
If you have any questions, you can contact a member of the research team by email at soham.bandyopadhyay@st-hildas.ox.ac.uk

Thank you for your consideration.

Many thanks,
<Your name>

SMART team

Appendix S4

Thank you for taking part in this research study. This information is intended for anyone who may be concerned or distressed after participating in this study. If this applies to you, I would like to point out that there are several sources of advice or help which are free and readily available to you and which may prove useful. Specifically, these include (please note, you may need to copy and paste the web addresses into your browser):

Your GP: Book an appointment with your GP. They can offer advice or refer you to other more specific services to get help.

Your University Counselling Service (where available)

NHS 111

FRANK: For friendly, confidential drugs advice, call FRANK on: 0300 123 6600 (0800776600 in Scotland) . Or, you can access their website at: http://www.talktofrank.com

MIND: Mind is a mental health charity serving England and Wales. To ask about mental health or mental health services, or to find out more about a particular Mind service, phone the MIND Information Line on 0300 123 3393. Trained staff and volunteers will be able to give you details of services in your area and talk through options with you.
https://www.mind.org.uk/

Students Against Depression: An award-winning website offering information, blogs and resources to help students find their way forward from low mood or depression: http://www.studentsagainstdepression.org

Student Minds: Student Minds is a national student mental health charity working to encourage peer interventions for student mental health. For more information, take a look at their website at: http://www.studentminds.org.uk

The Samaritans: Trained volunteers are able to listen to you any time day or night. We can help you talk through whatever is troubling you, find the answers that are right for you, and offer support. Call on: 116 123
https://www.samaritans.org/

LGBT foundation: a national charity delivering advice, support and information services to lesbian, gay, bisexual and trans (LGBT) communities
https://lgbt.foundation/

IN AN EMERGENCY: If you are experiencing suicidal thoughts and think that you might be unable to keep yourself safe, visit your nearest A&E department or call 999.

Appendix S5

Survey of Medical student Attitudes to Research and Training pathways (SMART) study

Thank you for your interest in participating in this study. Please take a moment to read the following information, before ticking the box to confirm your participation in the study.

If you have any questions or concerns, please contact the principal researcher, Soham Bandhyopadhyay, at soham.bandyopadhyay@st-hildas.ox.ac.uk

- **What is the aim of this study?** This study aims to ascertain current medical student involvement with research. We also hope to identify factors encouraging and discouraging students from partaking in research and to consider what may encourage more engagement with scientific research in the future.
- **Why have I been selected to take part?** You are being invited to take part in the questionnaire as you are a medical student currently studying for a UK medical degree at a UK medical school recognised by the General Medical Council (GMC).
- **What do I have to do?** If you choose to participate in this voluntary survey, you will be asked to complete a questionnaire about your background, your previous exposure to research and your feelings towards a research career. This study is voluntary. If you decide not to participate this will not impact your academic standing in any way. If you decide to take part, you will be asked to complete the survey by clicking on the link below. This survey is expected to take about 10 -15 minutes to complete, but there is no time limit and you can take as much time as you like. No background knowledge is required. We will ask for your consent for the collection and storage of data in accordance with the UK General Data Protection Regulation (GDPR) within the survey. For more information on GDPR please click on the following link: https://gdpr-info.eu/.
- **Do I have to participate?** Please note that your participation is voluntary. You may withdraw at any point during the questionnaire for any reason, before submitting your answers, by closing the browser. In cases of withdrawal from the study, no new data will be collected or linked to other data from that point on. If you do not want to answer some of the questions you do not have to, but you can still be in the study. All questions are optional. Your decision whether or not to be part of the study will not affect your academic standing or your access to university support services. If you have already submitted data and wish to withdraw from the study, please contact soham.bandyopadhyay@st-hildas.ox.ac.uk by 1st October 2021.
- **Who has approved this study?** This project has received ethics clearance through the University of Oxford’s ethical approval process for research involving human participants, reference R73479/RE001.
- **How will my data be used?** Your answers will be completely anonymous, and we will take all reasonable measures to ensure that they remain confidential. Your data will be stored in a password-protected file and may be

used in academic publications. Your IP address will not be stored. If you provide us with your email address, we will delete that information at the end of the study. No answers will be linked to your email address. Research data – your anonymised answers – will be stored for a minimum of ten years after publication or public release.

- **Who will have access to my data?** Qualtrics is the data controller with respect to the personal data they hold about you and, as such, will determine how your personal data is used. Please see their privacy notice here: https://www.qualtrics.com/privacy-statement. Qualtrics will share any email address you provide and your anonymised answers with the University of Oxford, for the purposes of research. Researchers involved in the project will have access to this anonymised data. The University of Oxford is the data controller of university email addresses, please see their privacy notice here: https://compliance.admin.ox.ac.uk/student-privacy-policy. Responsible members of the University of Oxford and funders may be given access to data for monitoring and/or audit of the study to ensure we are complying with guidelines, or as otherwise required by law.
- **Are there any benefits to taking part?** Despite not have any immediate individual benefits by participating in this survey, you are given the opportunity to contribute to valuable and innovative research which could be used in the future by medical universities and the world. You may find this survey an opportunity to self-reflect. There will be the option to submit email address in order to be entered into a prize draw. At the conclusion of data collection, two random participants will be awarded £50 in Amazon vouchers, two further random participants will be awarded £25 in Amazon vouchers. This will be optional, as it requires you to provide personally identifying data (i.e. contact details). These will not be linked to the questionnaire answers given, and will only be used for contact regarding relevant rewards as above.
- **Will the research be published?** The findings of the study may be published in peer reviewed journals, presented at relevant conferences and meetings and a summary of the findings will be made available on social media**.**
- **Are there any possible risks involved with my participation?** Some of the questions that we ask may cause upset. If you experience any distress from participating in this study, you may stop the survey at any time or skip any upsetting questions. If your distress continues after leaving the survey, we have provided a list of supportive services nationwide that can be helpful and that you might consider contacting (appendix S5, to be linked here, and appear again at the close of the survey).
- **Who do I contact if I have a concern about the study or I wish to complain?** If you have a concern about any aspect of this project, please speak to the researcher soham.bandyopadhyay@st-hildas.ox.ac.uk who will do their best to answer your query. The researchers should acknowledge your concern within 10 working days and give you an indication of how they intend to deal with it. If you remain unhappy or wish to make a formal complaint, please contact the Chair of the Medical Sciences Inter-Divisional Research Ethics Committee: Email: ethics@medsci.ox.ac.uk; Address: Research Services, University of Oxford, Wellington Square, Oxford OX1 2JD OR The Chair will seek to resolve the matter in a reasonably expeditious manner.

- **How do I find out what was learned in this study?** This study is expected to be completed by approximately March 2022. If you would like a brief summary of the results, please write to us by email to request information
- **Who to contact for further details?** For any further questions or more information on the study, please contact us on the following email address: soham.bandyopadhyay@st-hildas.ox.ac.uk. Alternatively, you could contact principal investigator Dr Catherine Swales at catherine.swales@ndorms.ox.ac.uk.

Please note that you may only participate in this survey if you are 18 years of age or over.
☐ I certify that I am 18 years of age or over

If you have read the information above and agree to participate with the understanding that the data (including any personal data) you submit will be processed accordingly, please check the relevant box below to get started.
☐ Yes, I agree to take part

Tick this box if you would like to be considered for the prize draw, and are happy to be contacted via your email address for such
☐ Yes, I would like to be entered into the draw

<u>Appendix S6</u>

Many thanks for filling out the SMART questionnaire. We ask you to kindly give us a further few minutes of your time to answer questions regarding the questionnaire experience in order to improve it for future users.

Approximately how many minutes did it take you to complete the questionnaire? *Numerical*
In your opinion was the questionnaire *too long/ just right/ too short*?
Did you find the questions clear and easy to understand? *Yes/No*
Does the questionnaire omit any issues or factors you consider important to investigate? *Yes/No with white space*
Did you have any problems completing the questionnaire? *White space*
Is there anything else you would like us to know about the questionnaire, or the distribution of the questionnaire? *White space*

<u>Appendix S7</u>
We will utilise the test of parallel lines, and the output of our Multiple Correspondence Analysis to test initial statistical assumptions. We may relax the assumptions of the model by allowing partial proportional odds, or a generalised ordinal logit to ensure valid statistical analysis. We have used the directed acyclic graph approach to minimize bias introduction for causal inference from our predictive model, through selective covariate and predictor variable selection.

Our proposed initial model is: **Going to pursue academic career = (Ethnicity + Gender + Medical School + Free school meals + 1st Degree relatives in academia + Year of study + LGBTQ+) (**Equation 1).

Equation 1.

$$logit[P(\text{Going to pursue academic career} \leq j)] = \alpha_j + \beta_{Ethnicity}X_{Ethnicity} + \beta_{Gender}X_{Gender} + \beta_{Medical\ School}X_{Medical\ School} + \beta_{Free\ School\ Meals}X_{Free\ School\ Meals} + \beta_{Relatives\ in\ academia}X_{Relatives\ in\ academia} + \beta_{Year\ of\ study}X_{Year\ of\ study} + \beta_{LGBTQ+}X_{LGBTQ+}.$$

Where 'Desire to pursue academic career' represents answers to Question 20. (How much do you agree with the statement: "I wish to pursue an academic career or an academic training pathway ."). j represents the number of possible answers to Question 20 – 1. Ethnicity represents answers to Question 4. (Choose one option that best describes your ethnic group or background?) Gender represents answers to Question 5 (What gender do you identify as?). Medical School represents answers to Question 1a (Which medical school do you attend? (If currently intercalating at a separate university, please give the university from which you will receive your main degree)). Free School Meals represents answers to Question 7. (During School, at any point, were you eligible for free school meals?). Relatives in academia represents answers to Question 9. (How many of your first degree relatives are or have been in academia?). Year of study represents answers to Question 1b. (What year of medical school are you currently in?). LGBTQ+ represents answers to Question 6. (Do you identify as LGBTQ+?).

We will utilize likelihood ratios, Akaike information criterion, Schwarz criterion, -2 log likelihood, Pseudo-R^2 (Cox-Snell) to ascertain if this model has acceptable fit. If this full model does not, then we will optimise for a single model out of the set of possible nested models which will then be used as our subsequent model. We will utilise sampling weights identified in 1.5.

<u>Appendix S8</u>

Our proposed initial model is: **Research in future = (Ethnicity + Gender + Medical School + Free school meals + 1st Degree relatives in academia + Year of study + LGBTQ+) (**Equation 2).

Equation 2.

$$logit[P(\text{Desire to research in future} \leq j)] = \alpha_j + \beta_{Ethnicity}X_{Ethnicity} + \beta_{Gender}X_{Gender} + \beta_{Medical\ School}X_{Medical\ School} + \beta_{Free\ School\ Meals}X_{Free\ School\ Meals} + \beta_{Relatives\ in\ academia}X_{Relatives\ in\ academia} + \beta_{Year\ of\ study}X_{Year\ of\ study} + \beta_{LGBTQ+}X_{LGBTQ+}.$$

Where the above variables represent the same as in Appendix S7, apart from Desire to research in the future represents answers to Question 21.

<u>Appendix S9</u>

Going to pursue academic career = (Ethnicity + Gender + Medical School + Free school meals + Why did research + previous Degree + Year of study + LGBTQ+) (Equation 3).

Equation 3.

$$\mathit{logit}[P(\mathit{Going\ to\ pursue\ academic\ career} \le j)] = \alpha_j + \beta_{Ethnicity}X_{Ethnicity} + \beta_{Gender}X_{Gender} + \beta_{Medical\ School}X_{Medical\ School} + \beta_{Free\ School\ Meals}X_{Free\ School\ Meals} + \beta_{Why\ did\ research}X_{Why\ did\ research} + \beta_{Previous\ degree}X_{Previous\ degree} + \beta_{Year\ of\ study}X_{Year\ of\ study} + \beta_{LGBTQ+}X_{LGBTQ+}.$$

Going to pursue academic career = (Ethnicity + Gender + Medical School + Free school meals + Barriers to voluntary research + Year of study + LGBTQ+) (Equation 4).

Equation 4.

$$\mathit{logit}[P(\mathit{Going\ to\ pursue\ academic\ career} \le j)] = \alpha_j + \beta_{Ethnicity}X_{Ethnicity} + \beta_{Gender}X_{Gender} + \beta_{Medical\ School}X_{Medical\ School} + \beta_{Free\ School\ Meals}X_{Free\ School\ Meals} + \beta_{Barriers\ to\ voluntary\ research}X_{Barriers\ to\ voluntary\ research} + \beta_{Year\ of\ study}X_{Year\ of\ study} + \beta_{LGBTQ+}X_{LGBTQ+}.$$

Where all previously defined variables are as defined in Appendix 7 or Appendix 8. ‘Why did research’ represents answers to Question 17. (Why did/do you do research?). ‘Previous degree’ represents answers to Question 2. (Have you already completed an academic degree?). Barriers to voluntary research represents the set of common themes identified in each answer to Question 15. (If you have not yet been involved in voluntary research, what have been the barriers preventing this?).]
