# Supplementary material for: Cross-sectional Survey of Medical student Attitudes to Research and Training pathways (SMART) in the UK: study protocol
Source: BMJ Open. 2021 Sep 2;11(9):e050104. doi: 10.1136/bmjopen-2021-050104 (PMC8413964; doi:10.1136/bmjopen-2021-050104)
Supplement: Author's manuscript [file bmjopen-2021-050104.draft_revisions.pdf]

BMJ Open

BMJ Open is committed to open peer review. As part of this commitment we make the peer review history of every article we publish publicly available.

When an article is published we post the peer reviewers' comments and the authors' responses online. We also post the versions of the paper that were used during peer review. These are the versions that the peer review comments apply to.

The versions of the paper that follow are the versions that were submitted during the peer review process. They are not the versions of record or the final published versions. They should not be cited or distributed as the published version of this manuscript.

BMJ Open is an open access journal and the full, final, typeset and author-corrected version of record of the manuscript is available on our site with no access controls, subscription charges or pay-per-view fees (http://bmjopen.bmj.com).

If you have any questions on BMJ Open's open peer review process please email info.bmjopen@bmj.com

BMJ Open

Survey of Medical student Attitudes to Research and Training pathways (SMART) Study Protocol

Journal:	*BMJ Open*
Manuscript ID	bmjopen-2021-050104
Article Type:	Protocol
Date Submitted by the Author:	11-Feb-2021

Complete List of Authors:	Roche, Sophie; University of Oxford, Medical Sciences Division<br>Bandyopadhyay, Soham; University of Oxford, Medical Sciences Division<br>Brown, Robin; University of Oxford, Medical Sciences<br>Eggington, Holly; University of Oxford, Medical Sciences Division<br>Iveson, Poppy; University of Oxford, Medical Sciences Division<br>Mallett, Garry; University of Oxford, Medical Sciences Division<br>Grassam-Rowe, Alexander; University of Oxford, Medical Sciences Division<br>Swales, Catherine; University of Oxford, Medical Sciences Division

Keywords:	MEDICAL EDUCATION & TRAINING, Protocols & guidelines < HEALTH SERVICES ADMINISTRATION & MANAGEMENT, QUALITATIVE RESEARCH

SCHOLARONE™
Manuscripts

I, the Submitting Author has the right to grant and does grant on behalf of all authors of the Work (as defined in the below author licence), an exclusive licence and/or a non-exclusive licence for contributions from authors who are: i) UK Crown employees; ii) where BMJ has agreed a CC-BY licence shall apply, and/or iii) in accordance with the terms applicable for US Federal Government officers or employees acting as part of their official duties; on a worldwide, perpetual, irrevocable, royalty-free basis to BMJ Publishing Group Ltd ("BMJ") its licensees and where the relevant Journal is co-owned by BMJ to the co-owners of the Journal, to publish the Work in this journal and any other BMJ products and to exploit all rights, as set out in our licence.

The Submitting Author accepts and understands that any supply made under these terms is made by BMJ to the Submitting Author unless you are acting as an employee on behalf of your employer or a postgraduate student of an affiliated institution which is paying any applicable article publishing charge ("APC") for Open Access articles. Where the Submitting Author wishes to make the Work available on an Open Access basis (and intends to pay the relevant APC), the terms of reuse of such Open Access shall be governed by a Creative Commons licence – details of these licences and which Creative Commons licence will apply to this Work are set out in our licence referred to above.

Other than as permitted in any relevant BMJ Author's Self Archiving Policies, I confirm this Work has not been accepted for publication elsewhere, is not being considered for publication elsewhere and does not duplicate material already published. I confirm all authors consent to publication of this Work and authorise the granting of this licence.

For peer review only

Survey of Medical student Attitudes to Research and Training pathways (SMART) Study Protocol

Sophie Roche[1], Soham Bandyopadhyay[1], Robin Brown[1], Holly Eggington[1], Poppy Iveson[1], Garry Mallett[1], Alexander Grassam-Rowe[1], Catherine Swales[1]

[1]Medical Sciences Division, University of Oxford, Oxford, UK

For peer review only

Corresponding author: Sophie Roche, Medical Sciences Office, Medical Sciences Division, John Radcliffe Hospital, Oxford, OX39DU sophie.roche@st-hughs.ox.ac.uk

Abstract

Objectives: This national, multi-centre study will use an online questionnaire focussing on medical student perceptions of research and research-orientated careers, including barriers and incentives to participating in research as a student and in following a research-orientated career.

Setting: The online questionnaire will be disseminated to all UK medical schools recognised by the GMC through collaborative university medical school and student networks.

Participants: All medical students currently studying for a UK medical degree at a UK medical school recognised by the General Medical Council (GMC) will be eligible to participate.

Primary and secondary outcome measures: The primary outcome is to ascertain perceptions of medical research amongst medical students studying at universities across the UK. Secondary outcomes include identifying the factors involved in incentivising and deterring medical students to do research during medical school, ascertaining whether different factors affect medical students at different stages of their training and establishing whether different background characteristics affect perceptions of medical research amongst medical students. We hope to discover how students have become involved in research and characterise the proportion of medical students who would want to be involved in research in their future career.

Results: The SMART study will determine the perceptions surrounding medical research amongst medical students studying at universities across the UK, and will identify personal and demographic factors both incentivising and deterring students from partaking in research while at medical school. It will also identify how students have become involved in research and characterise the proportion of students who would want to be involved in research in their future career.

Conclusions: This comprehensive study will provide information on current medical student views on academic career pathways.

Strengths and Limitations of this Study

- We will be the first to comprehensively examine the opinions of UK medical students towards research and training pathways
- This study engages students as collaborators and contributors to provide opportunity for interested students to be involved in a multicentre, national research project
- The design is limited by its snapshot nature, as we are unable to see how opinions have changed over time
- It is currently unclear whether there is a correlative or causative association between early career exposure to research opportunities and the later career paths taken.

For peer review only

Introduction

Clinical academics are a diverse group of doctors with roles in research and teaching alongside their clinical work; they are of vital importance to the progress of medical innovation as their combined academic and clinical perspectives allow the most pressing challenges in health care to be identified and studied. Their unique position affords an integrative outlook on research which provides possibilities of more cross-specialty and interdisciplinary work. In their teaching role, clinical academics also ensure that the curricula of medical schools are up-to-date with the latest research, and that students are instilled with the scientific rigour that is essential to becoming a good doctor in any specialty [1]. The perspectives and innovations of academic clinicians are of particular importance in the rapidly changing and pressurised modern health care environment. Such leadership is therefore required to ensure that the advances made in research are incorporated into clinical practice as efficiently as possible [2,3,4]. The next generation of clinical academics is essential to capture the medical benefit of the innovation sphere, and their number should be a key target to maintain and increase. Indeed, without clinical academics, we risk a ‘stagnation’ of our healthcare delivery [1].

Despite their clear importance, the total number of clinical academic staff employed by UK medical schools has declined since its peak in 2010 [5]. An insufficient number of individuals entering the clinical academic ‘pipeline’ has been identified as a contributing factor in this downward trend [5], resulting in reduced replacement of an aging workforce. This is surprising given that involvement in research is often seen as a desirable quality in the CVs of medical students and junior doctors [5,6]. Indeed, one survey of academic physicians found career success was independently associated with having conducted research as a student [7].

Given the need to maintain, and indeed increase, the workforce of clinical academics, it is important to identify the factors influencing medical students in deciding whether to engage in research. Previous data from other countries has identified intrinsic demographic factors which may affect interest in research, such as economic background, ethnicity, and gender. Non-demographic influences have also been identified, including previous involvement in research and access to mentors [8,9]. Similar data is however currently lacking for medical schools in the UK, and no clear data available for the interactions between demographic and non-demographic

influences. With variation in the ability for students to intercalate and medical school requirements for involvement in research during training [10], it is important that we understand the different opinions on clinical academia amongst UK medical students, the experiences that have shaped these, and the perceived barriers to pursuing research during medical school and beyond.

The Survey of Medical student Attitudes to Research and Training pathways (SMART) study is an online national questionnaire-based study. The aim of this study is to ascertain medical student perceptions of research and research-orientated careers, including barriers and incentives to participating in research as a student and in following a research-orientated career. It is our hope that increasing awareness of the issues medical students face will encourage solutions to be sought by medical schools and regulators who share the responsibility for developing future academic clinicians. SMART will also identify key issues for the Academy of Medical Sciences to address as part of their long-term INSPIRE strategy.

Methods and Analysis

Primary aim:

- To ascertain perceptions of medical research amongst medical students studying at universities across the UK

Secondary Aims:

- To identify the factors involved in incentivising medical students to do research during medical school
- To identify the factors involved in deterring medical students from doing research during medical school
- To ascertain whether different factors affect medical students at different stages of their training
- To ascertain whether different background characteristics affect perceptions of medical research amongst medical students
- To examine how different demographic effects interact with non-demographic effects
- To identify how students have become involved in research
- To characterise the proportion of medical students who would want to be involved in research in their future career
- To ascertain perceived incentives and barriers to becoming a clinical academic
- To identify scope for expanded integration of research within medical school curricula.

Study Design

SMART is an online, national, multi-centre, questionnaire-based study focussing on medical student perceptions of research and research-orientated careers, including barriers and incentives to participating in research as a student and in following a research-orientated career. The study will be disseminated through collaborative

For peer review only

university medical school and student networks, such as the network of INSPIRE leads across the country. The SMART study will be delivered by a team of University of Oxford medical students. The generic collaborative method, and the benefits that participating students derive from it are based on previous well-documented literature [11,12,13,14,15]. The questions included can be found in appendix S1.

Patient and public involvement

No patients or members of the public were involved in the design of the study. We will share results with interested individuals and publicly via journal publication.

Methods for recruiting participants

All medical students currently studying for a UK medical degree at a UK medical school recognised by the General Medical Council (GMC) will be eligible to participate. A list of these medical schools can be found in Appendix S2.

Medical students will be invited to participate in the study through several routes:

- Medical Societies
- Medical School mailing lists
- Organisations focused on academic medicine, such as the Academy of Medical Sciences
- Conferences
- Freshers' Fairs
- Social Media

In addition, medical students enrolled in the medical schools in Appendix S2 will be invited to collaborate in the study as regional leads as described in Table 1. The maximum number of collaborators from each medical school will be one per year. Appendix S3 details the participant facing information used in recruitment for the SMART study.

Collaborators will ensure that their medical school is formally engaged at an early stage of this study, and they will be primarily responsible for disseminating this questionnaire amongst students at their medical school. Medical school collaborators will be able to request for their own specific data and the analysis done on said data from the SMART steering committee following study completion. This data will be anonymised.

Dates	Activity
**1st March 2021 – 1st May 2021**	Study pilot to be run at Oxford
**1st April 2021 – 30th May 2021**	Recruit collaborators at all UK universities via collaboration with INSPIRE Student Leads

**1st June 2021 – 30th July 2021**	Study to be modified based on pilot feedback
**1st August 2021 – 1st September 2021**	Study set up e.g. training collaborators, gaining approval for this study at each centre, providing collaborators with Qualtrics logins
**1st September 2021 – 1st November 2021**	Study runs nationally for 2 months
**1st November 2021 – 31st December 2021**	Analyse data and prepare manuscript

Table 1: Project timeline. Extended data collection periods may be incorporated to grant flexibility to centres that may have experienced logistical obstacles to study commencement.

Information provided to participants

The following pieces of information will be provided to participants before taking part in the study. It will be attached to recruitment emails, and appear as the front page of the questionnaire:

- Name of the study: Survey of Medical student Attitudes to Research and Training pathways (SMART) study
- Name of the principal researcher carrying out the study and information on how to contact her: Sophie Roche sophie.roche@st-hughs.ox.ac.uk
- **What is the aim of this study?** This study aims to ascertain medical student perceptions of research and research-orientated careers, including barriers and incentives to participating in research as a student as well as following a research-orientated career.
- **Why have I been selected to take part?** You are being invited to take part in the questionnaire as you are a medical student currently studying for a UK medical degree at a UK medical school recognised by the General Medical Council (GMC).
- **What do I have to do?** If you choose to participate in this voluntary survey, you will be asked to complete a questionnaire about your background, your previous exposure to research and your feelings towards a research career. This study is voluntary. If you decide not to participate this will not impact your academic standing in any way. If you decide to take part, you will be asked to complete the survey by clicking on the link below. Each survey is expected to take about 10 -15 minutes to complete, but there is no time limit and you can take as much time as you like. No background knowledge is required. We will ask for your consent for the collection and storage of data in accordance with the UK General Data Protection Regulation (GDPR) within the survey. For more information on GDPR please click on the following link: https://gdpr-info.eu/.
- **Do I have to participate?** Please note that your participation is voluntary. You may withdraw at any point during the questionnaire for any reason, before submitting your answers, by closing the browser. In cases of

For peer review only - http://bmjopen.bmj.com/site/about/guidelines.xhtml

withdrawal from the study, no new data will be collected or linked to other data from that point on. If you do not want to answer some of the questions you do not have to, but you can still be in the study. As all questions are optional, we have included a 'Prefer not to answer' option for each set of questions. Your decision whether or not to be part of the study will not affect your academic standing or your access to university support services.

- **Who has approved this study?** This project has received ethics clearance through the University of Oxford's ethical approval process for research involving human participants, reference R73479/RE001.
- **How will my data be used?** Your answers will be completely anonymous, and we will take all reasonable measures to ensure that they remain confidential. Your data will be stored in a password-protected file and may be used in academic publications. Your IP address will not be stored. If you provide us with your email address, we will delete that information at the end of the study. No answers will be linked to your email address. Research data – your anonymised answers – will be stored for a minimum of ten years after publication or public release.
- **Who will have access to my data?** Qualtrics is the data controller with respect to the personal data they hold about you and, as such, will determine how your personal data is used. Please see their privacy notice here: https://www.qualtrics.com/privacy-statement. Qualtrics will share any email address you provide and your anonymised answers with the University of Oxford, for the purposes of research. The University of Oxford is the data controller of university email addresses, please see their privacy notice here: https://compliance.admin.ox.ac.uk/student-privacy-policy. Responsible members of the University of Oxford and funders may be given access to data for monitoring and/or audit of the study to ensure we are complying with guidelines, or as otherwise required by law.
- **Are there any benefits to taking part?** Despite not have any immediate individual benefits by participating in this survey, you are given the opportunity to contribute to valuable and innovative research which could be used in the future by medical universities and the world. You may find this survey an opportunity to self-reflect. There will be the option to submit email address in order to be entered into a prize draw. At the conclusion of data collection, two random participants will be awarded £50 in Amazon vouchers, two further random participants will be awarded £25 in Amazon vouchers. This will be optional, as it requires you to provide personally identifying data (i.e. contact details). These will not be linked to the questionnaire answers given, and will only be used for contact regarding relevant rewards as above.
- **Will the research be published?** The findings of the study may be published in peer reviewed journals, presented at relevant conferences and meetings and a summary of the findings will be made available on the website**.**
- **Are there any possible risks involved with my participation?** Some of the questions that we ask may cause upset or bring up painful memories. If you experience any distress from participating in this study, you may stop the survey at any time or skip any upsetting questions. If your distress continues after leaving the survey, we have provided a list of supportive services nationwide that can be helpful and that you might consider contacting (appendix S5, to be linked here, and appear again at the close of the survey).

For peer review only - http://bmjopen.bmj.com/site/about/guidelines.xhtml

- **Who do I contact if I have a concern about the study or I wish to complain?** If you have a concern about any aspect of this project, please speak to the researcher Sophie Roche at sophie.roche@st-hughs.ox.ac.uk who will do her best to answer your query. The researchers should acknowledge your concern within 10 working days and give you an indication of how they intend to deal with it. If you remain unhappy or wish to make a formal complaint, please contact the Chair of the Medical Sciences Inter-Divisional Research Ethics Committee: Email: ethics@medsci.ox.ac.uk; Address: Research Services, University of Oxford, Wellington Square, Oxford OX1 2JD OR The Chair will seek to resolve the matter in a reasonably expeditious manner.
- **How do I find out what was learned in this study?** This study is expected to be completed by approximately December 2021. If you would like a brief summary of the results, please write to us by email to request information
- **Who to contact for further details?** For any further questions or more information on the study, please contact us on the following email address: sophie.roche@st-hughs.ox.ac.uk. Alternatively, you could contact principal investigator Dr Catherine Swales at catherine.swales@ndorms.ox.ac.uk.

A representation of how this information will be presented to medical students can be found in Appendix S4.

Financial and other rewards to participants

A prize draw involving participants who have opted to provide their contact details will take place at the conclusion of data collection. Four random participants will be awarded £50 in Amazon vouchers. Two further random participants will be awarded £25 in Amazon vouchers..

Data Collection

The online questionnaire consists of 22 questions that utilise a combination of the Likert scale, multiple choice options and free text in order to broaden the capture and improve granularity of the data. The questionnaire has been sent to multiple medical students who were not involved in creating the data collection proforma. Their responses were evaluated for potential problems, and the questionnaire was updated to ensure the questions were relevant, comprehensive and accessible. A pilot study will be performed at Oxford Medical School before this study is launched nationally. There are 952 students enrolled at Oxford Medical School. Data collection will take place at the beginning of March 2021, aiming to be complete by May 2021. Based on the experiences from the pilot, the questionnaire will be modified to improve clarity, objectivity, and accuracy.

Primary Outcome

To find the predictors of whether an individual in our cohort intends to pursue an academic career.

Secondary Outcomes

For peer review only

The find the predictors of whether an individual in our cohort is interested in undertaking (more) research in the future.

Data management

All the data will be anonymised and stored in Qualtrics. Qualtrics is cloud-based platform, with the ability to create and customise databases. Qualtrics guarantees the highest levels of security for stored data and is compliant with regulations including GDPR and ICH E6 Good Clinical Practice. Users will have individual accounts, with access to data determined by the study administrator. Audit logs are also available to provide an overview of data access and modifications.

Statistical Analysis

What do we hope to ascertain:

Q1: What predicts whether an individual in our cohort intends to pursue an academic career?

Q2: What predicts whether an individual in our cohort is interested in undertaking (more) research in the future?

Statistical procedure:

Unless explicitly stated otherwise, assume alpha=0.05, and CI = 95%.

1. Data processing for descriptive statistics:

1. Data will be cleaned of any conflicting, impossible, or corrupted data by deletion of all entries of the same individual.
2. Percentages for responses by each question.
3. Median and deciles of spread for answers to appropriate question(s).
4. Mode for answers to appropriate question(s).
5. Number of missing values per question.
6. Number of individuals with 1 or more missing values.
7. Identify sampling weights for demographic populations utilizing data from local sources, and national data collected about medical student demographics at each school.
8. Key qualitative themes identified with free writing texts of questions 22 and 27.

2. Establishing correlation across responses.

1. Multiple Correspondence Analysis utilizing sampling weights from 1.7, with orthogonal polynomials to account for ordinality in data [14], where appropriate, to produce a simple visualisation of correlation across variables. All interval/ratio variables will be appropriately binned to produce ordinal variables for the analysis above. If a low number of responses then answers to question 4 may be binned into broader ethnicities and backgrounds, to maintain statistic power.

3. Testing Q1: What predicts whether an individual in our cohort intends to pursue an academic career?

For peer review only

We will run an ordinal logistic regression, using the cumulative logit link function. If we identify that certain variables display a high degree of co-linearity, we may deign to use a partial proportional odds model to produce the best model. We will use the ordinal responses to question 20. 'How much do you agree with the statement: "I wish to pursue an academic career." ' (Likert scale of "Disagree" through to "Strongly agree".) "), as our response variable, and plot a model with the answers to all questions as predictor variables, being a mixture of interval, ratio, ordinal, and nominal. We will utilise sampling weights identified in 1.5. We will assess a full model utilizing all variables, and then assess nested models, as to ascertain the best model fit. We will utilize likelihood ratios, Akaike information criterion, Schwarz criterion, -2 log likelihood, Pseudo-R^2 (Cox-Snell) to ascertain the model with the best fit, which will then be used as our subsequent model.

We will report the analyses of maximum likelihood estimates, and the odds ratio estimates.

<u>4. Q2: What predicts whether an individual in our cohort is interested in undertaking (more) research in the future?</u>

We will run an ordinal logistic regression, using the cumulative logit link function. If we identify that certain variables display a high degree of co-linearity, we may deign to use a partial proportional odds model to produce the best model. We will use the ordinal responses to question 21. How strongly do you agree with the statement: "I would be interested in undertaking (more) research in the future." (Likert scale of "Disagree" through to "Strongly agree".) as our response variable, with answers to all questions as predictor variables, being a mixture of interval, ratio, ordinal, and nominal. We will utilise sampling weights identified in 1.5. We will assess a full model utilizing all variables, and then assess nested models, as to ascertain the best model fit. We will utilize likelihood ratios, Akaike information criterion, Schwarz criterion, -2 log likelihood, and Pseudo-R^2 (Cox-Snell), to ascertain the model with the best fit, which will then be used as our subsequent model. We will report the analyses of maximum likelihood estimates, and the proportional odds ratio estimates.

Additional exploratory analysis may be carried out as deemed necessary, but it will be reported as such, and will be carried out with good statistical reporting in mind.

To ascertain whether there are any similar respondent segments, to help guide potential intervention, we will also attempt K-nearest neighbour clustering [17] to identify similarities across variables and respondents. If we are unable to find suitable distance metrics then we will attempt to implement a metric learning process with 80% of data points set aside for training, and 20% for testing.

Authorship

In accordance with National Research Collaborative (NRC) authorship guidelines [15], all publication outputs from SMART will be listed under a unified corporate authorship: 'SMART Collaborative'. Certain publications will include named authors on the bye-line as well as the group name. This will follow the example set by commendable collaboratives including STARSurg and InciSioN UK [16,17]. Anyone

For peer review only

who has demonstrated satisfactory completion of the minimum requirements for authorship will be eligible for PubMed-citable collaborative authorship in accordance with the roles defined below:

Writing Group: Responsible for the overall scientific content, data analysis, and preparation of research manuscripts.

Steering Committee: Responsible for the protocol design, project coordination, and data handling.

Collaborators: A network of medical students across all medical schools. They are responsible for leading the study regionally.

Ethics and dissemination

Ethics

Ethics approval has been obtained from Medical Sciences Interdivisional Research Ethics Committee, Oxford, England .

Dissemination

The protocol will be disseminated primarily through recruited medical student collaborators. Should UK medical schools wish to see the protocol, collaborators may pass it along as well. The study protocol will also be submitted for peer-reviewed publication. Any publications of the protocol will be advertised through social media, including Twitter and Facebook.

Following study completion, teleconferences will be held with all collaborators to share and discuss the data analysis undertaken and the study results. Following this, the results will be presented at local, regional, national, and international conferences by medical student collaborators. A standard PowerPoint presentation and poster will be created for this purpose. All presentations will be coordinated by the SMART steering committee to avoid duplications and to ensure all conference regulations are fulfilled. In addition, the results will be disseminated via publication in a peer-reviewed medical journal. All collaborators will be given PubMed citable collaborative co-authorship under the institutional name 'SMART Collaborative'. We will have a hybrid authorship list of named authors and the institutional collaborative.

Following publication, the manuscript can be shared by collaborators with their medical schools to feedback the study results, and to highlight the scope for expanded integration of research within medical school curricula. Medical schools can request for their own specific data and the analysis done on said data from the GHEMS steering committee following study completion. The fully anonymised dataset will be made publicly available.

Word count: 3774

For peer review only

References

1. Academy of Medical Sciences Working Party Report. The tenure-track clinician scientist: a new career pathway to promote recruitment into clinical academic medicine. *AMS* 2000.

2. Rees, M., Dangerfield, P., Katz, D., & Schofield, M. Every Doctor a Scientist and a Scholar. *BMA Publications 2015.*

3. BMA. The Role of the Clinical Academic. *BMA Publications 2014.*

4. Savill J. More in expectation than in hope: a new attitude to training in clinical academic medicine. *BMJ 2000*; 320:630

5. Medical Schools Council. Survey of medical clinical academic staffing levels 2017. www.medschools.ac.uk/SiteCollectionDocuments/Survey-Medical-Clinical-Academic-Staffing-Levels-2017.pdf.

6. Chambler AF, Chapman-Sheath PJ, Pearse MF. A model curriculum vitae: what are the trainers looking for? *Hospital Medicine 1998*;59:324–26.

7. Brancati FL, Mead LA, Levine DM et al. Early predictors of career achievement in academic medicine. *JAMA. 1992*;267(10):1372-6.

8. Mina S, Mostafa S, Albarqawi HT et al. Perceived influential factors towards participation in undergraduate research activities among medical students at Alfaisal University-College of Medicine: A Saudi Arabian perspective. *Med Teach. 2016*;38 :S31-6

9. Ejaz K, Shamim MS, Shamim MS, et al. Involvement of medical students and fresh medical graduates of Karachi, Pakistan in research. *J Pak Med Assoc. 2011*;61(2):115-20.

10. Nicholson JA, Cleland J, Lemon J. Why medical students choose not to carry out an intercalated BSc: a questionnaire study. *BMC Med Educ 2010*;10:25-6920-10-25.

11. National Research Collaborative & Association of Surgeons in Training Collaborative Consensus Group. Recognising contributions to work in research collaboratives: Guidelines for standardising reporting of authorship in collaborative research. *Int J Surg. 2018*;52:355-60.

12. Bhangu A, Fitzgerald JE, Kolias AG. Trainee-led research collaboratives: a novel model for delivering multi-centre studies. *ANZ J Surg.2014*;84(12):902-3.

13. Chapman SJ, Glasbey JC, Khatri C, et al. Promoting research and audit at medical school: evaluating the educational impact of participation in a student-led national collaborative study. *BMC Med Educ. 2015*;15(1):47.

14. Moon, K.R., van Dijk, D., Wang, Z. *et al.* Visualizing structure and transitions in high-dimensional biological data. *Nat Biotechnol 2019*: 1482–1492.

15. National Research Collaborative & Association of Surgeons in Training Collaborative Consensus Group. Recognising contributions to work in research collaboratives:

For peer review only

Guidelines for standardising reporting of authorship in collaborative research. *Int J Surg. 2018*;52:355-60.

16. STARSurg Collaborative. Prognostic model to predict postoperative acute kidney injury in patients undergoing major gastrointestinal surgery based on a national prospective observational cohort study. *BJS Open. 2018*;2(6):400-10.

17. Brancati FL, Mead LA, Levine DM et al. Early predictors of career achievement in academic medicine. *JAMA. 1992*;267(10):1372-6.

Authors' contributions

The study concept and design was conceived by SB and SR. SR, SB, RB, HE, PI and GM contributed equally to the preparation of the first draft of the manuscript. AG-R provided the statistical planning, analysis, and contributed to survey design. All authors provided edits and critiqued the manuscript

Funding

This work was supported by an INSPIRE grant, supported by the Wellcome Trust

Competing interests

None declared

For peer review only

Appendix S1

Proforma of questionnaire, to be converted to digital format for administration. Text in red describes the available answers for each question.

Q1. Which year of medical school are you currently in? (Ordinal)

☐ Please select which year of your medical degree you are currently enrolled in (First/Second/Third/Intercalation/Fourth/Fifth/Sixth/ Graduate Entry first/ Graduate Entry second/ Graduate Entry third/ Graduate Entry fourth

Q2. Have you already completed an academic degree (Bachelor's/Masters/Doctorate)? (binary)

☐ Please select whether you currently already have a completed higher education degree, such as a Bachelor's, Master's, or Doctorate. (YES/NO)

Q3. If you answered yes to Question 6, please select all those degrees you currently have. (Nominal/Ordinal)

☐ Please select all degrees you currently have. (Bachelor's – in a scientific degree/Bachelor's – in an arts degree/Master's – in a scientific degree/Master's – in an arts degree/Doctorate)

Q4. Choose one option that best describes your ethnic group or background? (Nominal)

☐ Please select which ethnic group you most strongly identify as. (English, Welsh, Scottish, Northern Irish, British/Irish/Gypsy or Irish Traveller/Any other White background/ White and Black Carribean/ White and Black African/ White and Asian/ Any other Mixed or Multiple ethnic background/ Indian/ Pakistani/ Bangladeshi/ Chinese/ Any other Asian background/ African/Caribbean/ Any other Black, African, or Caribbean background/Arab/ Any other ethnic group)

*Note, list taken from government list of ethnic groups https://www.ethnicity-facts-figures.service.gov.uk/style-guide/ethnic-groups

Q5. What gender do you identify as? (Nominal)

☐ Please select your gender from the options. (Female/Male/Non-Binary)

Q6. Do you identify as LGBTQ+? (Binary)

Please select whether you identify as LGBTQ+ (Lesbian, Gay, Bisexual, Transgender, Queer/Questioning, or any other part of the LGBTQ+ community). (YES/NO)

Q7. During school, at any point, were you eligible for free school meals? (binary)

Please select whether you were, at any point, eligible for free school meals during school. (Yes/No)

Q8. How many of your first degree relatives are or have been a healthcare professional? (Ratio/Ordinal)

Please select the number of relatives who currently or have ever identify/identified as healthcare professionals. (0,1,2,3,4,5,6,7,8,9,10,10+)

Q9. How many of your first degree relatives are or have been in academia? (Ratio/ordinal)

Please select the number of relatives who currently or have ever identify/identified as being in academia. (0,1,2,3,4,5,6,7,8,9,10,10+)

Q10. If you answered >0 for both Question 8 and Question 9, how many of your relatives are or have been academics in the healthcare environment? (Ratio/ordinal)

Please select all the number of relatives who currently or have ever identify/identified as academics in the healthcare environment. (0,1,2,3,4,5,6,7,8,9,10,10+)

Q11. In which area did you undertake the majority of your pre-university education?

Please select the area where you undertook the majority of your education before university (UK, EU, outside EU)

Q12. How well do you feel your medical school has educated you about research? (Ordinal)

Please select which best represents your opinion. (Not well at all/ Less than adequately/Adequately/More than adequately/Very well)

Q13. How much research have you undertaken to date? (Ordinal)

For peer review only

Please select how much research you feel you have undertaken to date. (None/A little bit/ A fair amount/Quite a lot/A very significant body of research)

Q14. If you have undertaken any research, was any of this a compulsory part of your degree? (Binary)

Please select whether any research you have done included a compulsory part of your degree. (Yes/No)

Q15. If you have not yet been involved in non-compulsory research, what have been the barriers preventing this? (Nominal)

Please the barriers that have prevented you from undertaking research outside of any compulsory degree obligation. (Free text up to 500 words)

Q16. Please tick any of the following that apply to your research: (Nominal?)

Please tick any of the following that apply to your research: (QIP/audit/basic science project/ clinical project/ co-author on original paper in peer reviewed journal/ co-author on any publications related to research/ named collaborator on original paper in peer reviewed journal/ named collaborator on any publication related to research/ presented a poster/ given an oral presentation)

Q17. Why did/do you do research? (Nominal)

Please tick any of the following that apply to your research:. (Interest in scientific problems/Interest in the subject/Personal development/Extra income/contribution to better health care/Improving critical thinking/Career progression/Intellectual stimulation/Feel obliged to do it/Other

Q18. How much do you think research is useful in combination with your medical studies? (Ordinal)

For peer review only

☐ Please select to what degree you feel research is useful in combination with your medical studies. (Not at all useful/ Somewhat useful/ A little useful/A lot useful/Very useful)

Q19. How difficult is it to combine research with your medical studies? (Ordinal)

☐ Please select how difficult you find it to integrate research with your medical studies. (Not at all difficult/Somewhat difficult/A little difficult/ A lot difficult/ Very difficult)

Q20. How much do you agree with the statement: "I wish to pursue an academic career." (Ordinal)

☐ Please select how strongly you intend to pursue an academic career. (Strongly disagree/ Somewhat disagree/No opinion/Somewhat agree/Strongly agree)

Q21. How much do you agree with the statement: "I would be interested in undertaking (more) research in the future." (Ordinal)

☐ Please select how strongly you agree with the above statement. (Strongly disagree/ Somewhat disagree/No opinion/Somewhat agree/Strongly agree)

Q22. What would encourage your involvement in research in the future? (Ordinal)

☐ Please tick all that apply
(More time/More incentives/ Easier access to research groups and projects/ Clearer information about how to get involved/ Clearer information about benefits of research/ Guaranteed rewards e.g. publication or presentation/ Other

<u>Appendix S2</u>
A list of medical schools recognised by the GMC as of 1st January 2020:
The University of Aberdeen
The University of Birmingham
The University of Bristol
The University of Buckingham
The University of Cambridge
Cardiff University
The University of Dundee
The University of East Anglia
The University of Edinburgh
University of Exeter Medical School
The University of Glasgow
The Imperial College of Science, Technology and Medicine

Keele University
King's College London
Lancaster University
The University of Leeds
The University of Leicester
The University of Liverpool
The University of London
The University of Manchester
The University of Newcastle
The University of Nottingham
The University of Oxford
Plymouth University Peninsula Schools of Medicine and Dentistry
Queen Mary University of London
The Queen's University of Belfast
St George's Hospital Medical School
Swansea University
The University of Sheffield
The University of Southampton
University College London
The University of Warwick
A combination of the University of Brighton and the University of Sussex
A combination of the University of Hull and the University of York

<u>Appendix S3</u>

This document outlines the participant facing information used in recruitment for the SMART study

1. **Enrolment and 1st Survey Phase:** Link shared via email and social media
    a. Initiation: 1st – 14th September
2. **1st and 2nd Follow up for 1st Survey**
    a. 14th September – 30th October
3. **3rd Follow up for 1st Survey**
    a. 31st October – 1st November

**Simplified Study Timeline**	
**1. Enrolment and 1st Survey Phase**	Initiation: 1st – 14th September
**2. First and Second Follow up for 1st Survey**	14th September – 30th October
**4. Third Follow up for 1st Survey**	31st October – 1st November

1. Initial email to students

Dear Students,

For peer review only

We are a group of researchers from across the country who are interested in understanding the medical student perceptions of research and research-orientated careers.

We invite you to participate in a 15-minute voluntary survey that is collecting information on your views on research and research-orientated careers. When you access the survey, you are going to be asked a variety of questions about your background, your previous experiences of research, and your thoughts on a future research career. Your IP address will not be recorded. All answers will remain anonymous and confidential.

If you wish to partake in our prize draw, please provide your email address when requested by the survey. This email address will not be linked to your answers. More information on the risks and benefits of completing this survey are included in the survey link. **You can access the survey here:**

<INS LINK>

If you have any questions, you can contact a member of the research team by email at sophie.roche@st-hughs.ox.ac.uk

Thank you for your consideration.

Many thanks,
<Your name>
SMART team

2. First and Second Follow-up email for 1st survey

Dear Students,

Just a friendly reminder that the SMART survey will remain open until November 1st, 2021. Please take a few moments to participate in this survey if you have not done so yet.

We invite you to participate in a 15-minute voluntary survey that is collecting information on your views on research and research-orientated careers. When you access the survey, you are going to be asked a variety of questions about your background, your previous experiences of research, and your thoughts on a future research career. Your IP address will not be recorded. All answers will remain anonymous and confidential.

If you wish to partake in our prize draw, please provide your email address when requested by the survey. This email address will not be linked to your answers. More information on the risks and benefits of completing this survey are included in the survey link. **You can access the survey here:**

<INS LINK>
If you have any questions, you can contact a member of the research team by email at sophie.roche@st-hughs.ox.ac.uk

For peer review only

Thank you for your consideration.

Many thanks,
<Your name>
SMART team

3. Third Follow-up email for 1st survey (sent out the day before the survey closes)

Dear Students,
Just a friendly reminder that the SMART survey will remain open until November 1st, 2021. Please take a few moments to participate in this survey if you have not done so yet.

We invite you to participate in a 15-minute voluntary survey that is collecting information on your views on research and research-orientated careers. When you access the survey, you are going to be asked a variety of questions about your background, your previous experiences of research, and your thoughts on a future research career. Your IP address will not be recorded. All answers will remain anonymous and confidential.

If you wish to partake in our prize draw, please provide your email address when requested by the survey. This email address will not be linked to your answers. More information on the risks and benefits of completing this survey are included in the survey link. **You can access the survey here:**

<INS LINK>
If you have any questions, you can contact a member of the research team by email at sophie.roche@st-hughs.ox.ac.uk

Thank you for your consideration.

Many thanks,
<Your name>
SMART team

Appendix S4

Survey of Medical student Attitudes to Research and Training pathways (SMART) study

Thank you for your interest in participating in this study. Please take a moment to read the following information, before ticking the box to confirm your participation in the study.

If you have any questions or concerns, please contact the principal researcher, Sophie Roche, at sophie.roche@st-hughs.ox.ac.uk.

What is the aim of this study?

For peer review only

This study aims to ascertain medical student perceptions of research and research-orientated careers, including barriers and incentives to participating in research as a student as well as following a research-orientated career.

Why have I been selected to take part?
You are being invited to take part in the questionnaire as you are a medical student currently studying for a UK medical degree at a UK medical school recognised by the General Medical Council (GMC).

What do I have to do?
If you choose to participate in this voluntary survey, you will be asked to complete a questionnaire about your background, your previous exposure to research and your feelings towards a research career. This study is voluntary. If you decide not to participate this will not impact your academic standing in any way. If you decide to take part, you will be asked to complete the survey by clicking on the link below. Each survey is expected to take about 10 -15 minutes to complete, but there is no time limit and you can take as much time as you like. No background knowledge is required.

We will ask for your consent for the collection and storage of data in accordance with the General Data Protection Regulation (GDPR) within the survey. For more information on GDPR please click on the following link: https://gdpr-info.eu/.

Do I have to participate?
Please note that your participation is voluntary. You may withdraw at any point during the questionnaire for any reason, before submitting your answers, by closing the browser. If you wish to discontinue from this study, you are free to do so at any time by not participating in any future questionnaires. In cases of withdrawal, no new data will be collected or linked to other data from that point on. If you do not want to answer some of the questions you do not have to, but you can still be in the study. As all questions are optional, we have included a 'Prefer not to answer' option for each set of questions. Your decision whether or not to be part of the study will not affect your academic standing or your access to university support services.

Who has approved this study?
This project has received ethics clearance through the University of Oxford's ethical approval process for research involving human participants.

How will my data be used?
Your answers will be completely anonymous, and we will take all reasonable measures to ensure that they remain confidential. Your data will be stored in a password-protected file and may be used in academic publications. Your IP address will not be stored. If you provide us with your email address, we will delete that information at the end of the study. No answers will be linked to your email address. Research data – your anonymised answers – will be stored for a minimum of ten years after publication or public release.
Who will have access to my data?
Qualtrics is the data controller with respect to the personal data they hold about you and, as such, will determine how your personal data is used. Please see their privacy notice here: https://www.qualtrics.com/privacy-statement. Qualtrics will

For peer review only

share any email address you provide and your anonymised answers with the University of Oxford, for the purposes of research. The University of Oxford is the data controller of university email addresses, please see their privacy notice here: https://compliance.admin.ox.ac.uk/student-privacy-policy. Responsible members of the University of Oxford and funders may be given access to data for monitoring and/or audit of the study to ensure we are complying with guidelines, or as otherwise required by law.

Are there any benefits to taking part?

Despite not have any immediate individual benefits by participating in this survey, you are given the opportunity to contribute to valuable and innovative research which could be used in the future by medical universities and the world. You may find this survey an opportunity to self-reflect.

There will be the option to submit contact details in order to be entered into a prize draw. At the conclusion of data collection, two random participants will be awarded £50 in Amazon vouchers, two further random participants will be awarded £25 in Amazon vouchers.

This will be optional, as it requires you to provide personally identifying data (i.e. contact details). These will not be linked to the questionnaire answers given, and will only be used for contact regarding relevant rewards as above.

Will the research be published?

The findings of the study may be published in peer reviewed journals, presented at relevant conferences and meetings and a summary of the findings will be made available on the website.

Are there any possible risks involved with my participation?

Some of the questions that we ask may cause upset or bring up painful memories. If you experience any distress from participating in this study, you may stop the survey at any time or skip any upsetting questions. If your distress continues after leaving the survey, we have provided a list of supportive services nationwide that can be helpful and that you might consider contacting.

Who do I contact if I have a concern about the study or I wish to complain?

If you have a concern about any aspect of this project, please speak to the researcher Sophie Roche at sophie.roche@st-hughs.ox.ac.uk who will do her best to answer your query. The researchers should acknowledge your concern within 10 working days and give you an indication of how they intend to deal with it. If you remain unhappy or wish to make a formal complaint, please contact the Chair of the Medical Sciences Inter-Divisional Research Ethics Committee: Email: ethics@medsci.ox.ac.uk; Address: Research Services, University of Oxford, Wellington Square, Oxford OX1 2JD OR

The Chair will seek to resolve the matter in a reasonably expeditious manner.

How do I find out what was learned in this study?

For peer review only

This study is expected to be completed by approximately December 2021. If you would like a brief summary of the results, please write to us by email to request information

Who to contact for further details?

For any further questions or more information on the study, please contact us on the following email address: sophie.roche@st-hughs.ox.ac.uk. Alternatively, you could contact principal investigator Dr Catherine Swales at catherine.swales@ndorms.ox.ac.uk.

Please note that you may only participate in this survey if you are 18 years of age or over.
☐ I certify that I am 18 years of age or over

If you have read the information above and agree to participate with the understanding that the data (including any personal data) you submit will be processed accordingly, please check the relevant box below to get started.
☐ Yes, I agree to take part

Tick this box if you would like to be considered for the prize draw, and are happy to be contacted via your email address for such
☐ Yes, I would like to be entered into the draw

Appendix S5

Thank you for taking part in this research study. This information is intended for anyone who may be concerned or distressed after participating in this study. If this applies to you, I would like to point out that there are several sources of advice or help which are free and readily available to you and which may prove useful. Specifically, these include (please note, you may need to copy and paste the web addresses into your browser):

Your GP: Book an appointment with your GP. They can offer advice or refer you to other more specific services to get help.

Your University Counselling Service (where available)

NHS 111

FRANK: For friendly, confidential drugs advice, call FRANK on: 0300 123 6600. Or, you can access their website at: http://www.talktofrank.com

MIND: Mind is a mental health charity. To ask about mental health or mental health services, or to find out more about a particular Mind service, phone the MIND Information Line on 01865 263730. Trained staff and volunteers will be able to give you details of services in your area and talk through options with you.
https://www.mind.org.uk/

For peer review only

Students Against Depression: An award-winning website offering information, blogs and resources to help students find their way forward from low mood or depression: http://www.studentsagainstdepression.org

Student Minds: Student Minds is a national student mental health charity working to encourage peer interventions for student mental health. For more information, take a look at their website at: http://www.studentminds.org.uk

The Samaritans: Trained volunteers are able to listen to you any time day or night. We can help you talk through whatever is troubling you, find the answers that are right for you, and offer support. Call on: 116 123
https://www.samaritans.org/

LGBT foundation: a national charity delivering advice, support and information services to lesbian, gay, bisexual and trans (LGBT) communities https://lgbt.foundation/

The Mental Health Crisis Line: 01865 251152

IN AN EMERGENCY: If you are experiencing suicidal thoughts and think that you might be unable to keep yourself safe, visit your nearest A&E department or call 999.

For peer review only

A Cross-Sectional Survey of Medical student Attitudes to Research and Training pathways (SMART) in the UK; Study Protocol

Journal:	*BMJ Open*
Manuscript ID	bmjopen-2021-050104.R1
Article Type:	Protocol
Date Submitted by the Author:	26-May-2021

Complete List of Authors:	Roche, Sophie; University of Oxford, Medical Sciences Division<br>Bandyopadhyay, Soham; University of Oxford, Medical Sciences Division<br>Grassam-Rowe, Alexander; University of Oxford, Medical Sciences Division<br>Brown, Robin; University of Oxford, Medical Sciences<br>Iveson, Poppy; University of Oxford, Medical Sciences Division<br>Mallett, Garry; University of Oxford, Medical Sciences Division<br>Eggington, Holly; University of Oxford, Medical Sciences Division<br>Swales, Catherine; University of Oxford, Medical Sciences Division

**Primary Subject Heading**:	Medical education and training
Secondary Subject Heading:	Research methods
Keywords:	MEDICAL EDUCATION & TRAINING, Protocols & guidelines < HEALTH SERVICES ADMINISTRATION & MANAGEMENT, QUALITATIVE RESEARCH, EDUCATION & TRAINING (see Medical Education & Training)

SCHOLARONE™
Manuscripts

I, the Submitting Author has the right to grant and does grant on behalf of all authors of the Work (as defined in the below author licence), an exclusive licence and/or a non-exclusive licence for contributions from authors who are: i) UK Crown employees; ii) where BMJ has agreed a CC-BY licence shall apply, and/or iii) in accordance with the terms applicable for US Federal Government officers or employees acting as part of their official duties; on a worldwide, perpetual, irrevocable, royalty-free basis to BMJ Publishing Group Ltd ("BMJ") its licensees and where the relevant Journal is co-owned by BMJ to the co-owners of the Journal, to publish the Work in this journal and any other BMJ products and to exploit all rights, as set out in our licence.

The Submitting Author accepts and understands that any supply made under these terms is made by BMJ to the Submitting Author unless you are acting as an employee on behalf of your employer or a postgraduate student of an affiliated institution which is paying any applicable article publishing charge ("APC") for Open Access articles. Where the Submitting Author wishes to make the Work available on an Open Access basis (and intends to pay the relevant APC), the terms of reuse of such Open Access shall be governed by a Creative Commons licence – details of these licences and which Creative Commons licence will apply to this Work are set out in our licence referred to above.

Other than as permitted in any relevant BMJ Author's Self Archiving Policies, I confirm this Work has not been accepted for publication elsewhere, is not being considered for publication elsewhere and does not duplicate material already published. I confirm all authors consent to publication of this Work and authorise the granting of this licence.

For peer review only

A Cross-Sectional Survey of Medical student Attitudes to Research and Training pathways (SMART) in the UK; Study Protocol

Sophie Roche[1], Soham Bandyopadhyay[1], Alexander Grassam-Rowe[1], Robin Brown[1],Poppy Iveson[1], Garry Mallett[1], Holly Eggington[1], Catherine Swales[1]

[1]Medical Sciences Division, University of Oxford, Oxford, UK

For peer review only

Corresponding author: Sophie Roche, Medical Sciences Office, Medical Sciences Division, John Radcliffe Hospital, Oxford, OX39DU sophie.l.roche@googlemail.com

Abstract

Background

Clinical academics are a diverse group of doctors with roles in research and teaching alongside their clinical work. Their value in the education of trainee doctors, and within the health service more broadly, is well documented. Despite this, the numbers of academic clinical staff has been falling since 2010. The medical schools council has previously reported that this decline has been influenced by a difficulty in recruiting trainees to research posts. To explore the reasons for this difficulty, this multicentre, cross-sectional study aims to determine medical student involvement and perceptions of research and research-orientated careers. It will additionally identify perceived barriers and incentives to participating in research as a student.

Methods and Analysis

This cross-sectional study of medical students at UK medical schools recognised by the General Medical Council will be administered using an online questionnaire. This will be disseminated nationally over a 2-month period through collaborative university medical school and student networks. The primary outcome is to determine the extent to which medical students are currently involved in research. Secondary outcomes include identifying the personal and demographic factors involved in incentivising and deterring medical students from becoming involved in research during medical school. This will be achieved using a selection of Likert scale, multiple choice and free text questions Ordinal logistic regression analysis will be performed to understand the association between specific factors and student involvement in research. This study will also characterise the proportion of medical students who are currently interested in conducting research in the future.

Ethics and dissemination

Ethics approval has been obtained from the Medical Sciences Interdivisional Research Ethics Committee, Oxford, England. The results will be disseminated via publication in a peer-reviewed medical journal and may be presented at local, regional, national, and international conferences by medical student collaborators.

Strengths and Limitations of this Study

- We will be the first to examine the attitudes of current UK medical students towards previous and future research opportunities
- The design is limited by its snapshot nature, as we are unable to see how attitudes have or will change over time
- It is possible that those already interested in this area will be more likely to complete the survey

Introduction

Clinical academics are a diverse group of doctors with roles in research and teaching alongside their clinical work; they are of vital importance to the progress of medical innovation as their combined academic and clinical perspectives allow the most pressing challenges in health care to be identified and studied. Their unique position affords an integrative outlook on research which provides possibilities of more cross-specialty and interdisciplinary work. In their teaching role, clinical academics also ensure that the curricula of medical schools are up-to-date with the latest research, and that students are instilled with the scientific rigour that is essential to becoming a good doctor in any specialty [1]. The perspectives and innovations of academic clinicians are of particular importance in the rapidly changing and pressurised modern health care environment. Such insight is therefore required to ensure that the advances made in research are incorporated into clinical practice as efficiently as possible [2,3,4]. The next generation of clinical academics is essential to capture the medical benefit of the innovation sphere, and their number should be a key target to maintain and increase. Indeed, without clinical academics, we risk a 'stagnation' of our healthcare delivery [1].

Despite their clear importance, the total number of clinical academic staff employed by UK medical schools has declined since its peak in 2010 [5]. An insufficient number of individuals entering the clinical academic 'pipeline' has been identified as a contributing factor in this downward trend [5], resulting in reduced replacement of an aging workforce. This is surprising given that involvement in research is often seen as a desirable quality in the CVs of medical students and junior doctors [6]. Indeed, one survey of academic physicians found career success was independently associated with having conducted research as a student [7] . Furthermore it has been shown that intercalation can have positive academic consequences [8] . Additionally, it is a GMC requirement for students to have a working knowledge of research [9], and requires clinical academics to teach them such.

While there have been attempts to survey the barriers and enablers of progression for early career clinical academics [10,11] there are currently no published systematic surveys of medical students which seek their views on academia. Furthermore, recent changes to the centralised application system has meant that research publications and additional degrees are no longer granted points as of 2023 [12,13], despite such points having been shown to be one of the primary motivating factors for medical student involvement in research [14,15]. This change has proved controversial [13,15] and several authors have raised concerns that fewer medical students will now become involved with research.

For peer review only

Given the need to maintain, and indeed increase, the workforce of clinical academics, it is important to identify the factors influencing medical students in deciding whether to engage in research. Previous studies have identified intrinsic demographic factors which may affect interest in particular specialty training programmes, including academic training pathways, and research, such as economic background, ethnicity, and gender [16,17]. Non-demographic influences have also been identified, including previous involvement in research and access to mentors [16,17,18]. Similar data is however currently lacking for medical schools in the UK, and no clear data available for the interactions between demographic and non-demographic influences. With variation in both medical school requirements for involvement in research during training and opportunities to intercalate [19], it is important that we understand the different attitudes towards clinical academia amongst UK medical students. We also hope to describe the experiences that have conceivably shaped these, and the perceived barriers to pursuing research during medical school and beyond.

The Survey of Medical student Attitudes to Research and Training pathways (SMART) study is an online national questionnaire-based study. The aim of this study is to ascertain medical student perceptions of research and research-orientated careers, including perceived barriers and incentives to participating in research as a student and in following a research-orientated career. It is our hope that increasing awareness of the issues medical students face will encourage solutions to be sought by medical schools and regulators who share the responsibility for developing future academic clinicians. SMART will also identify key issues for the Academy of Medical Sciences to address as part of their long-term INSPIRE strategy [20].

Methods and Analysis

Primary aim:

- To determine current medical students' involvement with research

Secondary Aims:

- To characterise the group of students who are interested in research
- To identify the factors that drive medical students to conduct research
- To identify the perceived barriers that have prevented current medical students from partaking in research
- To determine if current medical students are interested in conducting research
- To identify factors that could encourage current medical students to conduct research

Study Design

SMART is an online, national, multi-centre, questionnaire-based study focussing on medical student perceptions of research and research-orientated careers, including barriers and incentives to participating in research as a student and in following a

For peer review only

research-orientated career. The questionnaire will be disseminated through collaborative university medical school and student networks, such as the network of INSPIRE leads across the country. It is well documented that the generic collaborative method works, and that participating students benefit from involvement [21,22,23,24,25,26]. In short the collaborative method involves a ‘snap-shot, protocol-driven, pragmatic multicentre research’ (National Research Collaborative and Association of Surgeons in Training Collaborative Consensus Group 2018 p355), approach undertaken by separate groups of trainees, or in this case students; the collaborative method also allows for greater size and power of studies [21]. The SMART study will be delivered by a team of University of Oxford medical students in clinical years 4,5 and 6. The questions included can be found in appendix S1. The questions were formulated by medical students at Oxford University, with two students separately suggesting questions before being considered by all authors. Feedback will be collected from a pilot study regarding the suitability of questions and suggestions for other aspects to consider.

Patient and public involvement

No patients or members of the public were involved in the design of the study. We will share results with interested individuals and publicly via journal publication.

Methods for recruiting participants

All medical students currently studying for a UK medical degree at a UK medical school recognised by the General Medical Council (GMC) will be eligible to participate. A list of these medical schools can be found in Appendix S2.

Medical students will be invited to participate in the study through several routes:

- Medical Societies
- Medical School mailing lists
- Organisations focused on academic medicine, such as the Academy of Medical Sciences
- Conferences
- Freshers’ Fairs
- Social Media

In addition, medical students enrolled in the medical schools in Appendix S2 will be invited to collaborate in the study as regional leads as described in Table 1. The maximum number of collaborators from each medical school will be one per year. Appendix S3 details the participant facing information used in recruitment for the SMART study.

Collaborators will ensure that their medical school is formally engaged at an early stage of this study, and they will be primarily responsible for disseminating this questionnaire amongst students at their medical school. Medical school collaborators will be able to request for their own specific data and the analysis done on said data

For peer review only - http://bmjopen.bmj.com/site/about/guidelines.xhtml

from the SMART steering committee following study completion. This data will be anonymised. Researchers will have access to this anonymous data.

Dates	Activity
**1st September 2021 – 1st October 2021**	Study pilot to be run at Oxford
**1st April 2021 – 30th May 2021**	Recruit collaborators at all UK universities via collaboration with INSPIRE Student Leads
**1st October 2021 – 30th November 2021**	Study to be modified based on pilot feedback
**1st September 2021 – 1st October 2021**	Study set up e.g. training collaborators, gaining approval for this study at each centre, providing collaborators with Qualtrics logins
**1st November 2021 – 1st January 2022**	Study runs nationally for 2 months
**1st January 2022 – 31st March 2022**	Analyse data and prepare manuscript

Table 1: Project timeline. Extended data collection periods may be incorporated to grant flexibility to centres that may have experienced logistical obstacles to study commencement.

Information provided to participants

The following pieces of information will be provided to participants before taking part in the study. It will be attached to recruitment emails, and appear as the front page of the questionnaire:

- Name of the study: Cross-Sectional Survey of Medical student Attitudes to Research and Training pathways (SMART) study
- Name of the principal researcher carrying out the study and information on how to contact her: Sophie Roche sophie.l.roche@gmail.com
- **What is the aim of this study?** This study aims to ascertain current medical student involvement with research. We also hope to identify factors encouraging and discouraging students from partaking in research and to consider what may encourage more engagement with scientific research in the future.
- **Why have I been selected to take part?** You are being invited to take part in the questionnaire as you are a medical student currently studying for a UK medical degree at a UK medical school recognised by the General Medical Council (GMC).
- **What do I have to do?** If you choose to participate in this voluntary survey, you will be asked to complete a questionnaire about your background, your previous exposure to research and your feelings towards a research career. This study is voluntary. If you decide not to participate this will not impact your academic standing in any way. If you decide to take part, you will be asked to complete the survey by clicking on the link below. This survey is expected to take about 10 -15 minutes to complete, but there is no time limit and you can

take as much time as you like. No background knowledge is required. We will ask for your consent for the collection and storage of data in accordance with the UK General Data Protection Regulation (GDPR) within the survey. For more information on GDPR please click on the following link: https://gdpr-info.eu/.

- **Do I have to participate?** Please note that your participation is voluntary. You may withdraw at any point during the questionnaire for any reason, before submitting your answers, by closing the browser. In cases of withdrawal from the study, no new data will be collected or linked to other data from that point on. If you do not want to answer some of the questions you do not have to, but you can still be in the study. All questions are optional. Your decision whether or not to be part of the study will not affect your academic standing or your access to university support services. If you have already submitted data and wish to withdraw from the study, please contact sophie.l.roche@gmail.com by 1st October 2021.
- **Who has approved this study?** This project has received ethics clearance through the University of Oxford's ethical approval process for research involving human participants, reference R73479/RE001.
- **How will my data be used?** Your answers will be completely anonymous, and we will take all reasonable measures to ensure that they remain confidential. Your data will be stored in a password-protected file and may be used in academic publications. Your IP address will not be stored. If you provide us with your email address, we will delete that information at the end of the study. No answers will be linked to your email address. Research data – your anonymised answers – will be stored for a minimum of ten years after publication or public release.
- **Who will have access to my data?** Qualtrics is the data controller with respect to the personal data they hold about you and, as such, will determine how your personal data is used. Please see their privacy notice here: https://www.qualtrics.com/privacy-statement. Qualtrics will share any email address you provide and your anonymised answers with the University of Oxford, for the purposes of research. Researchers involved in the project will have access to this anonymised data. The University of Oxford is the data controller of university email addresses, please see their privacy notice here: https://compliance.admin.ox.ac.uk/student-privacy-policy. Responsible members of the University of Oxford and funders may be given access to data for monitoring and/or audit of the study to ensure we are complying with guidelines, or as otherwise required by law.
- **Are there any benefits to taking part?** Despite not have any immediate individual benefits by participating in this survey, you are given the opportunity to contribute to valuable and innovative research which could be used in the future by medical universities and the world. You may find this survey an opportunity to self-reflect. There will be the option to submit email address in order to be entered into a prize draw. At the conclusion of data collection, two random participants will be awarded £50 in Amazon vouchers, two further random participants will be awarded £25 in Amazon vouchers. This will be optional, as it requires you to provide personally identifying data (i.e. contact details). These will not be linked to the questionnaire answers given, and will only be used for contact regarding relevant rewards as above.

For peer review only

- **Will the research be published?** The findings of the study may be published in peer reviewed journals, presented at relevant conferences and meetings and a summary of the findings will be made available on social media**.**
- **Are there any possible risks involved with my participation?** Some of the questions that we ask may cause upset. If you experience any distress from participating in this study, you may stop the survey at any time or skip any upsetting questions. If your distress continues after leaving the survey, we have provided a list of supportive services nationwide that can be helpful and that you might consider contacting (appendix S4, to be linked here, and appear again at the close of the survey).
- **Who do I contact if I have a concern about the study or I wish to complain?** If you have a concern about any aspect of this project, please speak to the researcher Sophie Roche at sophie.l.roche@gmail.com who will do her best to answer your query. The researchers should acknowledge your concern within 10 working days and give you an indication of how they intend to deal with it. If you remain unhappy or wish to make a formal complaint, please contact the Chair of the Medical Sciences Inter-Divisional Research Ethics Committee: Email: ethics@medsci.ox.ac.uk; Address: Research Services, University of Oxford, Wellington Square, Oxford OX1 2JD OR The Chair will seek to resolve the matter in a reasonably expeditious manner.
- **How do I find out what was learned in this study?** This study is expected to be completed by approximately March 2022. If you would like a brief summary of the results, please write to us by email to request information
- **Who to contact for further details?** For any further questions or more information on the study, please contact us on the following email address: sophie.l.roche@gmail.com. Alternatively, you could contact principal investigator Dr Catherine Swales at catherine.swales@ndorms.ox.ac.uk.

A representation of how this information will be presented to medical students can be found in Appendix S5.

Financial and other rewards to participants

A prize draw involving participants who have opted to provide their contact details will take place at the conclusion of data collection. Two random participants will be awarded £50 in Amazon vouchers. Two further random participants will be awarded £25 in Amazon vouchers..

Data Collection

The online questionnaire consists of 22 quantitative and qualitative questions that utilise a combination of the Likert scale, multiple choice options and free text in order to broaden the capture of sentiment nuance and improve precision in the data. The questionnaire has been sent to medical students who were not involved in creating the data collection proforma. Their responses were evaluated for potential problems, and the questionnaire was updated to ensure the questions were relevant, comprehensive and accessible. A pilot study will be performed at Oxford Medical School before this study is launched nationally. There are 952 students enrolled at Oxford Medical School. Data collection will take place at the beginning of September

For peer review only

2021, aiming to be complete by November 2021. Based on the experiences from the pilot, the questionnaire will be modified to improve clarity, objectivity, and acceptability (appendix S6).

Primary Outcome

To describe the extent of current medical students' involvement with research

Secondary Outcomes

The identify and understand the reasons driving and excluding medical students from research. To identify factors that might encourage more current students to conduct research.

Data management

All the data will be anonymised and stored in Qualtrics. Qualtrics is cloud-based platform, with the ability to create and customise databases. Qualtrics guarantees the highest levels of security for stored data and is compliant with regulations including GDPR and ICH E6 Good Clinical Practice [27]. The research team will have individual accounts, with access to data determined by the study administrator. Audit logs are also available to provide an overview of data access and modifications.

Statistical Analysis

We will use descriptive statistics to achieve our primary outcome in describing the extent of current medical students' involvement with research.

In order to achieve our secondary outcomes we will consider the following research questions using machine learning techniques to predict in our cohort:
Q1: What is associated with whether an individual in our cohort intends to pursue an academic career?

Q2: What is associated with a respondent's interest in undertaking (more) research in the future?"

Statistical procedure:

Unless explicitly stated otherwise, assume alpha=0.05, and CI = 95%. Data will be cleaned of any conflicting, impossible, or corrupted data by deletion of all entries of the same individual.

1. Data processing for descriptive statistics:

This section seeks to provide an easy to interact with description of the responses to our survey, and the demographics of those who responded.

1. Percentages for responses by each question.
2. Median and deciles of spread for answers to appropriate question(s).
3. Mode for answers to appropriate question(s).

1. Number of missing values per question. Data will be assumed to be missing completely at random.
4. Number of individuals with 1 or more missing values.

5. Identify sampling weights for demographic populations utilizing data from local sources, and national data collected about medical student demographics at each school.
6. Key qualitative themes identified with free writing texts of questions 15, 16, 17, 22. We will look to identify common sentiments, having trained multiple different team members to independently look across the data and identify recurrent themes and sentiments. Each response will be evaluated by at least two of the trial team.

2. Establishing correlation across responses.

This section aims to find any predictive associations and identify likely explanatory variables. As a method, it also allows capture of the entirety of the cohort data.

We will perform Multiple Correspondence Analysis, utilizing sampling weights from 1.5. We will account for ordinality in data with orthogonal polynomials [28], where appropriate, to produce a simple visualisation of correlation across variables. All interval/ratio variables will be appropriately binned to produce ordinal variables for the analysis above. If there are a low number of responses then answers to question 4 may be binned into broader ethnicities and backgrounds, in order to maintain statistical power.

3. Testing Q1: What is associated with whether an individual in our cohort intends to pursue an academic career?

We will run an ordinal logistic regression, using the cumulative logit link function. We will use the ordinal responses to question 20. ‘How much do you agree with the statement: “I wish to pursue an academic career or an academic training pathway.” ’ (Likert scale of “Disagree” through to “Strongly agree”.) ”), as our response variable, and use as predictor variables the answers to other questions. Detail on proposed model construction can be found in appendix S7.

4. Q2: What is associated with a respondent's interest in undertaking (more) research in the future?"

The methods will be the same as above, using the ordinal responses to question 21. How strongly do you agree with the statement: “I would be interested in undertaking (more) research in the future.” (Likert scale of “Disagree” through to “Strongly agree”.) as our response variable, with answers to all questions as predictor variables as detailed in appendix S8 model construction.

5 Subgroup analysis.
With a priori assumptions about significant contributors to variance, one may attempt to control for these through subgroup analysis, in categorisation of the data points by a given variable or variables.

However, to ensure validity of causal inference, one must ensure that the appropriate covariates are selected for any regression model attempting to interrogate such data [29]. We believe a priori that we may see a large degree of variance between those who have already completed research, and those who have not. To account for this, we propose two models (appendix S9, equations 3 and 4) to

For peer review only

account for this difference and ascertain key contributors in these different groups, with the goal of more policy-relevant inferences being drawn for each subgroup.

Additional subgroup analysis may be carried out if key contributors to variance are identified in the Multiple Correspondence Analysis, or otherwise. Additional exploratory analysis may be carried out as deemed necessary, but it will be reported as such, and will be carried out with good statistical reporting in mind.

Authorship

In accordance with National Research Collaborative (NRC) authorship guidelines [24], all publication outputs from SMART will be listed under a unified corporate authorship: 'SMART Collaborative'. Certain publications will include named authors on the bye-line as well as the group name. This will follow the example set by commendable collaboratives including STARSurg and InciSioN UK [25,26]. Anyone who has demonstrated satisfactory completion of the minimum requirements for authorship will be eligible for PubMed-citable collaborative authorship in accordance with the roles defined below:

Writing Group: Responsible for the overall scientific content, data analysis, and preparation of research manuscripts.

Steering Committee: Responsible for the protocol design, project coordination, and data handling.

Collaborators: A network of medical students across all medical schools. They are responsible for leading the study regionally.

Ethics and dissemination

Ethics
Ethics approval has been obtained from Medical Sciences Interdivisional Research Ethics Committee, Oxford, England. Reference R73479/RE001.

Dissemination

The protocol will be disseminated primarily through recruited medical student collaborators. Should UK medical schools wish to see the protocol, collaborators may pass it along as well. Any publications of the protocol will be advertised through social media.

Following study completion, teleconferences will be held with all collaborators to share and discuss the data analysis undertaken and the study results. Following this, the results will be presented at local, regional, national, and international conferences by medical student collaborators. A standard PowerPoint presentation and poster will be created for this purpose. All presentations will be coordinated by the SMART steering committee to avoid duplications and to ensure all conference regulations are fulfilled. In addition, the results will be disseminated via publication in a peer-reviewed medical journal. All collaborators will be given PubMed citable

For peer review only

collaborative co-authorship under the institutional name 'SMART Collaborative'. We will have a hybrid authorship list of named authors and the institutional collaborative.

Following publication, the manuscript can be shared by collaborators with their medical schools to feedback the study results, and to highlight the scope for expanded integration of research within medical school curricula. Medical schools can request for their own specific data and the analysis done on said data from the steering committee following study completion. The fully anonymised dataset will be made publicly available.

Word count: 3968

References

1. Academy of Medical Sciences Working Party Report. The tenure-track clinician scientist: a new career pathway to promote recruitment into clinical academic medicine. *AMS* 2000.

2. Rees, M., Dangerfield, P., Katz, D., & Schofield, M. Every Doctor a Scientist and a Scholar. *BMA Publications 2015*.

3. BMA. The Role of the Clinical Academic. *BMA Publications 2014.*

4. Savill J. More in expectation than in hope: a new attitude to training in clinical academic medicine. *BMJ 2000*; 320:630

5. Medical Schools Council. Survey of medical clinical academic staffing levels 2017. www.medschools.ac.uk/SiteCollectionDocuments/Survey-Medical-Clinical-Academic-Staffing-Levels-2017.pdf.

6. Chambler AF, Chapman-Sheath PJ, Pearse MF. A model curriculum vitae: what are the trainers looking for? *Hospital Medicine 1998*;59:324–26.

7. Brancati FL, Mead LA, Levine DM et al. Early predictors of career achievement in academic medicine. *JAMA. 1992*;267(10):1372-6.

8. Mahesan N, Crichton S, Sewell H, Howell S. The effect of an intercalated BSc on subsequent academic performance. *BMC Med Educ*. 2011 Oct 3;11:76.

9. General Medical Council. Outcomes for Graduates. [online] Available at: https://www.gmc-uk.org/education/standards-guidance-and-curricula/standards-and-outcomes/outcomes-for-graduates/outcomes-for-graduates (Accessed May 2021)

10. Lopes J, Ranieri V, Lambert T, *et al.* The clinical academic workforce of the future: a cross-sectional study of factors influencing career decision-making among clinical PhD students at two research-intensive UK universities. *BMJ Open* 2017; **7:**e016823.

11. Oldfield K, Turner C, Garnett E. A Cross-Funder Review of Early-Career Clinical Academics: Enablers and Barriers to Progression. Available at <https://mrc.ukri.org/documents/pdf/review-of-early-career-clinical-academics/> [Accessed 12 May 2021].

12. *MSC Statement on the UKFPO decision to remove Educational Achievements from the Foundation ranking process | Medical Schools Council*. [online] Available at: <https://www.medschools.ac.uk/news/msc-statement-on-the-ukfpo-decision-to-remove-educational-achievements-from-the-foundation-ranking-process> [Accessed 12 May 2021].

13. British Medical Association. *Additional achievements ruled out of foundation programme applications*. [online] Available at: <https://www.bma.org.uk/news-and-opinion/additional-achievements-ruled-out-of-foundation-programme-applications> [Accessed 12 May 2021].

14. Pang KH, Hobbis C, Burleigh EJ, Miah S. Publication Component of the UK Foundation Programme Application: Perception of Medical Students. *Adv Med Educ Prac*t. 2020 Oct 8;11:735-740.

15. McVeigh J, Jeilani M, Super J. Factors influencing the engagement in research of medical undergraduates and clinical trainees. *Postgraduate Medical Journal* Published Online First: 16 February 2021.

16. Mina S, Mostafa S, Albarqawi HT et al. Perceived influential factors towards participation in undergraduate research activities among medical students at Alfaisal University-College of Medicine: A Saudi Arabian perspective. *Med Teach. 2016*;38 :S31-6

17. Amgad M, Man Kin Tsui M, Liptrott SJ, Shash E. Medical Student Research: An Integrated Mixed-Methods Systematic Review and Meta-Analysis. PLoS One. 2015 Jun 18;10(6):e0127470.

18. Ejaz K, Shamim MS, Shamim MS, et al. Involvement of medical students and fresh medical graduates of Karachi, Pakistan in research. *J Pak Med Assoc. 2011*;61(2):115-20.

19. Nicholson JA, Cleland J, Lemon J. Why medical students choose not to carry out an intercalated BSc: a questionnaire study. *BMC Med Educ 2010*;10:25-6920-10-25.

20. Acmedsci.ac.uk. 2021. *INSPIRE | The Academy of Medical Sciences*. [online] Available at: <https://acmedsci.ac.uk/grants-and-schemes/mentoring-and-other-schemes/INSPIRE> [Accessed 12 May 2021].

21. National Research Collaborative & Association of Surgeons in Training Collaborative Consensus Group. Recognising contributions to work in research collaboratives: Guidelines for standardising reporting of authorship in collaborative research. *Int J Surg. 2018*;52:355-60.

22. Bhangu A, Fitzgerald JE, Kolias AG. Trainee-led research collaboratives: a novel model for delivering multi-centre studies. *ANZ J Surg. 2014*;84(12):902-3.

23. Chapman SJ, Glasbey JC, Khatri C, et al. Promoting research and audit at medical school: evaluating the educational impact of participation in a student-led national collaborative study. *BMC Med Educ. 2015*;15(1):47.

24. National Research Collaborative & Association of Surgeons in Training Collaborative Consensus Group. Recognising contributions to work in research collaboratives:

For peer review only

Guidelines for standardising reporting of authorship in collaborative research. *Int J Surg. 2018*;52:355-60.

25. STARSurg Collaborative. Prognostic model to predict postoperative acute kidney injury in patients undergoing major gastrointestinal surgery based on a national prospective observational cohort study. *BJS Open. 2018*;2(6):400-10.

26. Bandyopadhyay S, Shortland T, Wadanamby SW, Thomas HS, Gurung B, Akhbari M, et al. Global Health Education in Medical Schools (GHEMS) study protocol. *J Glob Health Rep.* 2019; 3:e2019052.

27. European Medicines Agency. Available at <https://www.ema.europa.eu/en/documents/scientific-guideline/ich-e-6-r2-guideline-good-clinical-practice-step-5_en.pdf>. [Accessed 12 May 2021].

28. Rosaria Lombardo & Eric J. Beh (2010) Simple and multiple correspondence analysis for ordinal-scale variables using orthogonal polynomials, *Journal of Applied Statistics*, 37:12, 2101-2116, DOI: 10.1080/02664760903247692

29. Johannes Textor, Benito van der Zander, Mark S Gilthorpe, Maciej Liśkiewicz, George TH Ellison, Robust causal inference using directed acyclic graphs: the R package ‘dagitty’, *International Journal of Epidemiology*, Volume 45, Issue 6, December 2016, Pages 1887–1894

Authors’ contributions

The study concept and design was conceived by SB and SR. Abstract was written by GM, PI and SR. Strengths and limitations were written by SR. Introduction was written by SR, RB and HE. Methods were written by SR. Statistical analysis was written by SR and AG-R. AG-R provided the statistical planning, analysis, and contributed to survey design. . Authorship was written by SR. Ethics and dissemination was written by SR. CS and SB had a supervisory role at all stages of manuscript preparation, helped prepare and write funding applications and critically reviewed the manuscript. All authors critically reviewed the manuscript.

Funding

This work was supported by an INSPIRE grant from the Wellcome Trust [20]. No grant number is available.

Competing interests

None declared

For peer review only

Appendix S1

Proforma of questionnaire, to be converted to digital format for administration. Text in red describes the available answers for each question.

Q1a. Which medical school do you attend? (If currently intercalating at a separate university, please give the university from which you will receive your main degree).

☐ Please select your university from the dropdown list
as in appendix S2

Q1b. Which year of medical school are you currently in? (Ordinal)

☐ Please select which year of your medical degree you are currently enrolled in (First/Second/Third/ /Year 4 of 5/ Year 4 of 6/Year 5 of 6/ Year 5 of 5 /Year 6 of 6/ Intercalation/ Graduate Entry first/ Graduate Entry second/ Graduate Entry third/ Graduate Entry fourth

Q2. Have you already completed an academic degree (Bachelor's/Masters/Doctorate)? (binary)

☐ Please select whether you currently already have a completed higher education degree, such as a Bachelor's, Master's, or Doctorate. (YES/NO)

Q3. If you answered yes to Question 2, please select all those degrees you currently have. (Nominal/Ordinal)

☐ Please select all degrees you currently have. (Bachelor's – in a scientific degree/Bachelor's – in an arts degree/Master's – in a scientific degree/Master's – in an arts degree/Doctorate/ Other (please type))

Q4. Choose one option that best describes your ethnic group or background? (Nominal)

☐ Please select which ethnic group you most strongly identify as. (English, Welsh, Scottish, Northern Irish, British/Irish/Gypsy or Irish Traveller/Any other White background/ White and Black Carribean/ White and Black African/ White and Asian/ Any other Mixed or Multiple ethnic background/ Indian/ Pakistani/ Bangladeshi/ Chinese/ Any other Asian background/ African/Caribbean/ Any other Black, African, or Caribbean background/Arab/ Any other ethnic group)

*Note, list taken from government list of ethnic groups https://www.ethnicity-facts-figures.service.gov.uk/style-guide/ethnic-groups

Q5. What best describes your gender (Nominal)

Female/ Male/ Prefer not to say/ Prefer to self-describe (free text)

Q6. Do you identify as LGBTQ+? (Binary)

Please select whether you identify as LGBTQ+ (Lesbian, Gay, Bisexual, Transgender, Queer/Questioning, or any other part of the LGBTQ+ community). (YES/NO)

Q7. During school, at any point, were you eligible for free school meals? (binary)

Please select whether you were, at any point, eligible for free school meals during school. (Yes/No)

Q8. How many of your first degree relatives are or have been a healthcare professional*? (Ratio/Ordinal)

Please select the number of relatives who currently or have ever identify/identified as healthcare professionals. (0,1,2,3,4,5,6,7,8,9,10,10+)

*Healthcare professional defined according to WHO international classification of healthcare workers as professionals who study, advise on or provide preventive, curative, rehabilitative and promotional health services based on an extensive body of theoretical and factual knowledge in diagnosis and treatment of disease and other health problems. This includes:

- General medical practitioners
- Specialist medical practitioners
- Nursing professionals
- Midwifery professionals
- Traditional and complementary medicine professionals
- Paramedical practitioners
- Dentists
- Pharmacists
- Environmental and occupational health and hygiene professionals
- Physiotherapists

For peer review only

- Dieticians and nutritionists
- Optometrists and ophthalmic opticians

Q8b. If you answered >0 Question 8, how many of your relatives are or have been held an academic position* in the healthcare environment? (Ratio/ordinal)

☐ Please select all the number of relatives who currently or have ever identify/identified as academics in the healthcare environment. (0,1,2,3,4,5,6,7,8,9,10,10+)

*Academic position defined as working within a research or teaching capacity within a higher education institute. This includes, but is not limited, to, professors, readers, senior lecturers, lecturers, post-doctoral researchers, research fellows, teaching fellows

Q9. How many of your first degree relatives are or have been in academia? (Ratio/ordinal)

☐ Please select the number of relatives who currently or have ever identify/identified as being in academia. (0,1,2,3,4,5,6,7,8,9,10,10+)

Q9b. If you answered >0 Question 9, how many of your relatives are or have been held an academic position in the healthcare environment? (Ratio/ordinal)

☐ Please select all the number of relatives who currently or have ever identify/identified as academics in the healthcare environment. (0,1,2,3,4,5,6,7,8,9,10,10+)

*Academic position defined as working within a research or teaching capacity within a higher education institute. This includes, but is not limited, to, professors, readers, senior lecturers, lecturers, post-doctoral researchers, research fellows, teaching fellows

Q11. In which area did you undertake the majority of your pre-university education?

☐ Please select the geographical area where you undertook the majority of your education before university (UK, EU, outside EU)

For peer review only

Q12. How well do you feel your medical school has educated you about research? (Ordinal)

☐ Please select which best represents your opinion. (Not well at all/ Less than adequately/Adequately/More than adequately/Very well)

Q13. How much research have you undertaken to date? (Ordinal)

☐ Please select how much research you feel you have undertaken to date. (None/A little bit/ A fair amount/Quite a lot/A very significant body of research)

Q14. If you have undertaken any research, was any of this a compulsory part of your degree? (Binary)

☐ Please select whether any research you have done included a compulsory part of your degree. (Yes/No)

Q15. If you have not yet been involved in voluntary research, what have been the barriers preventing this? (Nominal)

☐ Please describe the barriers that have prevented you from undertaking research outside of any compulsory degree obligation. (Free text up to 500 words)

Q16. Please tick any of the following that apply to your research. You may tick more than one box or none at all : (Nominal)

☐ Please tick any of the following that apply to your research: (QIP/audit/basic science project/ clinical project/ co-author on original paper in peer reviewed journal/ co-author on any publications related to research/ named collaborator on original paper in peer reviewed journal/ named collaborator on any publication related to research/ presented a poster/ given an oral presentation/other (please type))

For peer review only

Q17. Why did/do you do research? (Nominal)

☐ Please tick any of the following that apply to your research:. (Interest in scientific problems/Interest in the subject/Personal development/Extra income/contribution to better health care/Improving critical thinking/Career progression/Intellectual stimulation/Feel obliged to do it/Other (please type)

Q18. How much do you think research is useful in combination with your medical studies? (Ordinal)

☐ Please select to what degree you feel research is useful in combination with your medical studies. (Not at all useful/ Somewhat useful/ A little useful/A lot useful/Very useful)

Q19. How difficult is it to combine research with your medical studies? (Ordinal)

☐ Please select how difficult you find it to integrate research with your medical studies. (Not at all difficult/Somewhat difficult/A little difficult/ A lot difficult/ Very difficult)

Q20. How much do you agree with the statement: "I wish to pursue an academic career or an academic training pathway ." (Ordinal)

☐ Please select how strongly you intend to pursue an academic career. (Strongly disagree/ Somewhat disagree/No opinion/Somewhat agree/Strongly agree)

Q21. How much do you agree with the statement: "I would be interested in undertaking (more) research in the future." (Ordinal)

☐ Please select how strongly you agree with the above statement. (Strongly disagree/ Somewhat disagree/No opinion/Somewhat agree/Strongly agree)

For peer review only

Q22. What would encourage your involvement in research in the future? (Ordinal)

☐ Please tick all that apply
(More time/More incentives/ Easier access to research groups and projects/ Clearer information about how to get involved/ Clearer information about benefits of research/ Guaranteed rewards e.g. publication or presentation/ Other (please type)

<u>Appendix S2</u>

A list of medical schools recognised by the GMC as of 1st January 2020:

The University of Aberdeen
The University of Birmingham
The University of Bristol
The University of Buckingham
The University of Cambridge
Cardiff University
The University of Dundee
The University of East Anglia
The University of Edinburgh
University of Exeter Medical School
The University of Glasgow
The Imperial College of Science, Technology and Medicine
Keele University
King’s College London
Lancaster University
The University of Leeds
The University of Leicester
The University of Liverpool
The University of London
The University of Manchester
The University of Newcastle
The University of Nottingham
The University of Oxford
Plymouth University Peninsula Schools of Medicine and Dentistry
Queen Mary University of London
The Queen’s University of Belfast
St George's Hospital Medical School
Swansea University
The University of Sheffield
The University of Southampton
University College London
The University of Warwick
A combination of the University of Brighton and the University of Sussex
A combination of the University of Hull and the University of York

For peer review only

Appendix S3

This document outlines the participant facing information used in recruitment for the SMART study

1. **Enrolment and 1st Survey Phase:** Link shared via email and social media
    a. Initiation: 1st – 14th November
2. **1st and 2nd Follow up for 1st Survey**
    a. 14th November – 30th November
3. **3rd Follow up for 1st Survey**
    a. 31st November – 1st January

1. Initial email to students

Dear Students,

We are a group of researchers from across the country who are interested in understanding the medical student perceptions of research and research-orientated careers.

We invite you to participate in a 15-minute voluntary survey that is collecting information on your views on research and research-orientated careers. When you access the survey, you are going to be asked a variety of questions about your background, your previous experiences of research, and your thoughts on a future research career. Your IP address will not be recorded. All answers will remain anonymous and confidential.

If you wish to partake in our prize draw, please provide your email address when requested by the survey. This email address will not be linked to your answers. More information on the risks and benefits of completing this survey are included in the survey link. **You can access the survey here:**

<INS LINK>

If you have any questions, you can contact a member of the research team by email at sophie.l.roche@gmail.com

Thank you for your consideration.

Many thanks,
<Your name>
SMART team

2. First and Second Follow-up email for 1st survey

Dear Students,

For peer review only

Just a friendly reminder that the SMART survey will remain open until January 1st, 2022. Please take a few moments to participate in this survey if you have not done so yet.

We invite you to participate in a 15-minute voluntary survey that is collecting information on your views on research and research-orientated careers. When you access the survey, you are going to be asked a variety of questions about your background, your previous experiences of research, and your thoughts on a future research career. Your IP address will not be recorded. All answers will remain anonymous and confidential.

If you wish to partake in our prize draw, please provide your email address when requested by the survey. This email address will not be linked to your answers. More information on the risks and benefits of completing this survey are included in the survey link. **You can access the survey here:**

<INS LINK>
If you have any questions, you can contact a member of the research team by email at sophie.l.roche@gmail.com

Thank you for your consideration.

Many thanks,
<Your name>
SMART team

3. Third Follow-up email for 1st survey (sent out the day before the survey closes)

Dear Students,
Just a friendly reminder that the SMART survey will remain open until January 1st, 2022. Please take a few moments to participate in this survey if you have not done so yet.

We invite you to participate in a 15-minute voluntary survey that is collecting information on your views on research and research-orientated careers. When you access the survey, you are going to be asked a variety of questions about your background, your previous experiences of research, and your thoughts on a future research career. Your IP address will not be recorded. All answers will remain anonymous and confidential.

If you wish to partake in our prize draw, please provide your email address when requested by the survey. This email address will not be linked to your answers. More information on the risks and benefits of completing this survey are included in the survey link. **You can access the survey here:**

<INS LINK>
If you have any questions, you can contact a member of the research team by email at sophie.l.roche@gmail.com

Thank you for your consideration.

For peer review only

Many thanks,
<Your name>
SMART team

Appendix S4

Thank you for taking part in this research study. This information is intended for anyone who may be concerned or distressed after participating in this study. If this applies to you, I would like to point out that there are several sources of advice or help which are free and readily available to you and which may prove useful. Specifically, these include (please note, you may need to copy and paste the web addresses into your browser):

Your GP: Book an appointment with your GP. They can offer advice or refer you to other more specific services to get help.

Your University Counselling Service (where available)

NHS 111

FRANK: For friendly, confidential drugs advice, call FRANK on: 0300 123 6600 (0800776600 in Scotland) . Or, you can access their website at: http://www.talktofrank.com

MIND: Mind is a mental health charity serving England and Wales. To ask about mental health or mental health services, or to find out more about a particular Mind service, phone the MIND Information Line on 0300 123 3393. Trained staff and volunteers will be able to give you details of services in your area and talk through options with you.
https://www.mind.org.uk/

Students Against Depression: An award-winning website offering information, blogs and resources to help students find their way forward from low mood or depression: http://www.studentsagainstdepression.org

Student Minds: Student Minds is a national student mental health charity working to encourage peer interventions for student mental health. For more information, take a look at their website at: http://www.studentminds.org.uk

The Samaritans: Trained volunteers are able to listen to you any time day or night. We can help you talk through whatever is troubling you, find the answers that are right for you, and offer support. Call on: 116 123
https://www.samaritans.org/

LGBT foundation: a national charity delivering advice, support and information services to lesbian, gay, bisexual and trans (LGBT) communities
https://lgbt.foundation/

For peer review only

IN AN EMERGENCY: If you are experiencing suicidal thoughts and think that you might be unable to keep yourself safe, visit your nearest A&E department or call 999.

Appendix S5

Survey of Medical student Attitudes to Research and Training pathways (SMART) study

Thank you for your interest in participating in this study. Please take a moment to read the following information, before ticking the box to confirm your participation in the study.

If you have any questions or concerns, please contact the principal researcher, Sophie Roche, at sophie.l.roche@gmail.com.

- **What is the aim of this study?** This study aims to ascertain current medical student involvement with research. We also hope to identify factors encouraging and discouraging students from partaking in research and to consider what may encourage more engagement with scientific research in the future.
- **Why have I been selected to take part?** You are being invited to take part in the questionnaire as you are a medical student currently studying for a UK medical degree at a UK medical school recognised by the General Medical Council (GMC).
- **What do I have to do?** If you choose to participate in this voluntary survey, you will be asked to complete a questionnaire about your background, your previous exposure to research and your feelings towards a research career. This study is voluntary. If you decide not to participate this will not impact your academic standing in any way. If you decide to take part, you will be asked to complete the survey by clicking on the link below. This survey is expected to take about 10 -15 minutes to complete, but there is no time limit and you can take as much time as you like. No background knowledge is required. We will ask for your consent for the collection and storage of data in accordance with the UK General Data Protection Regulation (GDPR) within the survey. For more information on GDPR please click on the following link: https://gdpr-info.eu/.
- **Do I have to participate?** Please note that your participation is voluntary. You may withdraw at any point during the questionnaire for any reason, before submitting your answers, by closing the browser. In cases of withdrawal from the study, no new data will be collected or linked to other data from that point on. If you do not want to answer some of the questions you do not have to, but you can still be in the study. All questions are optional. Your decision whether or not to be part of the study will not affect your academic standing or your access to university support services. If you have already submitted data and wish to withdraw from the study, please contact sophie.l.roche@gmail.com by 1st October 2021.
- **Who has approved this study?** This project has received ethics clearance through the University of Oxford's ethical approval process for research involving human participants, reference R73479/RE001.

For peer review only - http://bmjopen.bmj.com/site/about/guidelines.xhtml

- **How will my data be used?** Your answers will be completely anonymous, and we will take all reasonable measures to ensure that they remain confidential. Your data will be stored in a password-protected file and may be used in academic publications. Your IP address will not be stored. If you provide us with your email address, we will delete that information at the end of the study. No answers will be linked to your email address. Research data – your anonymised answers – will be stored for a minimum of ten years after publication or public release.
- **Who will have access to my data?** Qualtrics is the data controller with respect to the personal data they hold about you and, as such, will determine how your personal data is used. Please see their privacy notice here: https://www.qualtrics.com/privacy-statement. Qualtrics will share any email address you provide and your anonymised answers with the University of Oxford, for the purposes of research. Researchers involved in the project will have access to this anonymised data. The University of Oxford is the data controller of university email addresses, please see their privacy notice here: https://compliance.admin.ox.ac.uk/student-privacy-policy. Responsible members of the University of Oxford and funders may be given access to data for monitoring and/or audit of the study to ensure we are complying with guidelines, or as otherwise required by law.
- **Are there any benefits to taking part?** Despite not have any immediate individual benefits by participating in this survey, you are given the opportunity to contribute to valuable and innovative research which could be used in the future by medical universities and the world. You may find this survey an opportunity to self-reflect. There will be the option to submit email address in order to be entered into a prize draw. At the conclusion of data collection, two random participants will be awarded £50 in Amazon vouchers, two further random participants will be awarded £25 in Amazon vouchers. This will be optional, as it requires you to provide personally identifying data (i.e. contact details). These will not be linked to the questionnaire answers given, and will only be used for contact regarding relevant rewards as above.
- **Will the research be published?** The findings of the study may be published in peer reviewed journals, presented at relevant conferences and meetings and a summary of the findings will be made available on social media**.**
- **Are there any possible risks involved with my participation?** Some of the questions that we ask may cause upset. If you experience any distress from participating in this study, you may stop the survey at any time or skip any upsetting questions. If your distress continues after leaving the survey, we have provided a list of supportive services nationwide that can be helpful and that you might consider contacting (appendix S5, to be linked here, and appear again at the close of the survey).
- **Who do I contact if I have a concern about the study or I wish to complain?** If you have a concern about any aspect of this project, please speak to the researcher Sophie Roche at sophie.l.roche@gmail.com who will do her best to answer your query. The researchers should acknowledge your concern within 10 working days and give you an indication of how they intend to deal with it. If you remain unhappy or wish to make a formal complaint, please contact the Chair of the Medical Sciences Inter-Divisional Research Ethics Committee: Email: ethics@medsci.ox.ac.uk; Address: Research

For peer review only

Services, University of Oxford, Wellington Square, Oxford OX1 2JD OR The Chair will seek to resolve the matter in a reasonably expeditious manner.

- **How do I find out what was learned in this study?** This study is expected to be completed by approximately March 2022. If you would like a brief summary of the results, please write to us by email to request information
- **Who to contact for further details?** For any further questions or more information on the study, please contact us on the following email address: sophie.l.roche@gmail.com. Alternatively, you could contact principal investigator Dr Catherine Swales at catherine.swales@ndorms.ox.ac.uk.

Please note that you may only participate in this survey if you are 18 years of age or over.
☐ I certify that I am 18 years of age or over

If you have read the information above and agree to participate with the understanding that the data (including any personal data) you submit will be processed accordingly, please check the relevant box below to get started.
☐ Yes, I agree to take part

Tick this box if you would like to be considered for the prize draw, and are happy to be contacted via your email address for such
☐ Yes, I would like to be entered into the draw

Appendix S6

Many thanks for filling out the SMART questionnaire. We ask you to kindly give us a further few minutes of your time to answer questions regarding the questionnaire experience in order to improve it for future users.

Approximately how many minutes did it take you to complete the questionnaire? *Numerical*
In your opinion was the questionnaire *too long/ just right/ too short*?
Did you find the questions clear and easy to understand? *Yes/No*
Does the questionnaire omit any issues or factors you consider important to investigate? *Yes/No with white space*
Did you have any problems completing the questionnaire? *White space*
Is there anything else you would like us to know about the questionnaire, or the distribution of the questionnaire? *White space*

Appendix S7
We will utilise the test of parallel lines, and the output of our Multiple Correspondence Analysis to test initial statistical assumptions. We may relax the assumptions of the model by allowing partial proportional odds, or a generalised ordinal logit to ensure valid statistical analysis. We have used the directed acyclic graph approach to minimize bias introduction for causal inference from our predictive model, through selective covariate and predictor variable selection.

For peer review only

Our proposed initial model is: **Going to pursue academic career = (Ethnicity + Gender + Medical School + Free school meals + 1st Degree relatives in academia + Year of study + LGBTQ+) (**Equation 1).

Equation 1.

$$logit[P(Going\ to\ pursue\ academic\ career \leq j)] = \alpha_j + \beta_{Ethnicity}X_{Ethnicity} + \beta_{Gender}X_{Gender} + \beta_{Medical\ School}X_{Medical\ School} + \beta_{Free\ School\ Meals}X_{Free\ School\ Meals} + \beta_{Relatives\ in\ academia}X_{Relatives\ in\ academia} + \beta_{Year\ of\ study}X_{Year\ of\ study} + \beta_{LGBTQ+}X_{LGBTQ+}.$$

Where 'Desire to pursue academic career' represents answers to Question 20. (How much do you agree with the statement: "I wish to pursue an academic career or an academic training pathway ."). j represents the number of possible answers to Question 20 – 1. Ethnicity represents answers to Question 4. (Choose one option that best describes your ethnic group or background?) Gender represents answers to Question 5 (What gender do you identify as?). Medical School represents answers to Question 1a (Which medical school do you attend? (If currently intercalating at a separate university, please give the university from which you will receive your main degree)). Free School Meals represents answers to Question 7. (During School, at any point, were you eligible for free school meals?). Relatives in academia represents answers to Question 9. (How many of your first degree relatives are or have been in academia?). Year of study represents answers to Question 1b. (What year of medical school are you currently in?). LGBTQ+ represents answers to Question 6. (Do you identify as LGBTQ+?).

We will utilize likelihood ratios, Akaike information criterion, Schwarz criterion, -2 log likelihood, Pseudo-R^2 (Cox-Snell) to ascertain if this model has acceptable fit. If this full model does not, then we will optimise for a single model out of the set of possible nested models which will then be used as our subsequent model. We will utilise sampling weights identified in 1.5.

Appendix S8

Our proposed initial model is: **Research in future = (Ethnicity + Gender + Medical School + Free school meals + 1st Degree relatives in academia + Year of study + LGBTQ+) (**Equation 2).

Equation 2.

$$logit[P(Desire\ to\ research\ in\ future \leq j)] = \alpha_j + \beta_{Ethnicity}X_{Ethnicity} + \beta_{Gender}X_{Gender} + \beta_{Medical\ School}X_{Medical\ School} + \beta_{Free\ School\ Meals}X_{Free\ School\ Meals} + \beta_{Relatives\ in\ academia}X_{Relatives\ in\ academia} + \beta_{Year\ of\ study}X_{Year\ of\ study} + \beta_{LGBTQ+}X_{LGBTQ+}.$$

Where the above variables represent the same as in Appendix S7, apart from Desire to research in the future represents answers to Question 21.

Appendix S9

Going to pursue academic career = (Ethnicity + Gender + Medical School + Free school meals + Why did research + previous Degree + Year of study + LGBTQ+) (Equation 3).

Equation 3.

$$logit[P(\text{Going to pursue academic career} \leq j)] = \alpha_j + \beta_{Ethnicity}X_{Ethnicity} + \beta_{Gender}X_{Gender} + \beta_{Medical\ School}X_{Medical\ School} + \beta_{Free\ School\ Meals}X_{Free\ School\ Meals} + \beta_{Why\ did\ research}X_{Why\ did\ research} + \beta_{Previous\ degree}X_{Previous\ degree} + \beta_{Year\ of\ study}X_{Year\ of\ study} + \beta_{LGBTQ+}X_{LGBTQ+}.$$

Going to pursue academic career = (Ethnicity + Gender + Medical School + Free school meals + Barriers to voluntary research + Year of study + LGBTQ+) (Equation 4).

Equation 4.

$$logit[P(\text{Going to pursue academic career} \leq j)] = \alpha_j + \beta_{Ethnicity}X_{Ethnicity} + \beta_{Gender}X_{Gender} + \beta_{Medical\ School}X_{Medical\ School} + \beta_{Free\ School\ Meals}X_{Free\ School\ Meals} + \beta_{Barriers\ to\ voluntary\ research}X_{Barriers\ to\ voluntary\ research} + \beta_{Year\ of\ study}X_{Year\ of\ study} + \beta_{LGBTQ+}X_{LGBTQ+}.$$

Where all previously defined variables are as defined in Appendix 7 or Appendix 8. 'Why did research' represents answers to Question 17. (Why did/do you do research?). 'Previous degree' represents answers to Question 2. (Have you already completed an academic degree?). Barriers to voluntary research represents the set of common themes identified in each answer to Question 15. (If you have not yet been involved in voluntary research, what have been the barriers preventing this?).]

For peer review only

BMJ Open

A Cross-Sectional Survey of Medical student Attitudes to Research and Training pathways (SMART) in the UK; Study Protocol

Journal:	*BMJ Open*
Manuscript ID	bmjopen-2021-050104.R2
Article Type:	Protocol
Date Submitted by the Author:	28-Jun-2021
Complete List of Authors:	Roche, Sophie; University of Oxford, Medical Sciences Division<br>Bandyopadhyay, Soham; University of Oxford, Medical Sciences Division<br>Grassam-Rowe, Alexander; University of Oxford, Medical Sciences Division<br>Brown, Robin; University of Oxford, Medical Sciences<br>Iveson, Poppy; University of Oxford, Medical Sciences Division<br>Mallett, Garry; University of Oxford, Medical Sciences Division<br>Eggington, Holly; University of Oxford, Medical Sciences Division<br>Swales, Catherine; University of Oxford, Medical Sciences Division
**Primary Subject Heading**:	Medical education and training
Secondary Subject Heading:	Research methods
Keywords:	MEDICAL EDUCATION & TRAINING, Protocols & guidelines < HEALTH SERVICES ADMINISTRATION & MANAGEMENT, QUALITATIVE RESEARCH, EDUCATION & TRAINING (see Medical Education & Training)

SCHOLARONE™ Manuscripts

I, the Submitting Author has the right to grant and does grant on behalf of all authors of the Work (as defined in the below author licence), an exclusive licence and/or a non-exclusive licence for contributions from authors who are: i) UK Crown employees; ii) where BMJ has agreed a CC-BY licence shall apply, and/or iii) in accordance with the terms applicable for US Federal Government officers or employees acting as part of their official duties; on a worldwide, perpetual, irrevocable, royalty-free basis to BMJ Publishing Group Ltd ("BMJ") its licensees and where the relevant Journal is co-owned by BMJ to the co-owners of the Journal, to publish the Work in this journal and any other BMJ products and to exploit all rights, as set out in our licence.

The Submitting Author accepts and understands that any supply made under these terms is made by BMJ to the Submitting Author unless you are acting as an employee on behalf of your employer or a postgraduate student of an affiliated institution which is paying any applicable article publishing charge ("APC") for Open Access articles. Where the Submitting Author wishes to make the Work available on an Open Access basis (and intends to pay the relevant APC), the terms of reuse of such Open Access shall be governed by a Creative Commons licence – details of these licences and which Creative Commons licence will apply to this Work are set out in our licence referred to above.

Other than as permitted in any relevant BMJ Author's Self Archiving Policies, I confirm this Work has not been accepted for publication elsewhere, is not being considered for publication elsewhere and does not duplicate material already published. I confirm all authors consent to publication of this Work and authorise the granting of this licence.

For peer review only

A Cross-Sectional Survey of Medical student Attitudes to Research and Training pathways (SMART) in the UK; Study Protocol

Sophie Roche[1], Soham Bandyopadhyay[1], Alexander Grassam-Rowe[1], Robin Brown[1],Poppy Iveson[1], Garry Mallett[1], Holly Eggington[1], Catherine Swales[1]

[1]Medical Sciences Division, University of Oxford, Oxford, UK

Corresponding author: Sophie Roche, Medical Sciences Office, Medical Sciences Division, John Radcliffe Hospital, Oxford, OX39DU sophie.l.roche@googlemail.com

For peer review only

Abstract

Background

An understanding and appreciation of scientific research is a key quality of the modern clinician. Yet the medical schools council has previously reported a reduction in the number of clinicians performing research. To explore the reasons for this difficulty, this multicentre, cross-sectional study aims to determine medical student involvement and perceptions of research and research-orientated careers. It will additionally identify perceived barriers and incentives to participating in research as a student.

Methods and Analysis

This cross-sectional study of medical students at UK medical schools recognised by the General Medical Council will be administered using an online questionnaire. This will be disseminated nationally over a 2-month period through collaborative university medical school and student networks. The primary outcome is to determine the extent to which medical students are currently involved in research. Secondary outcomes include identifying the personal and demographic factors involved in incentivising and deterring medical students from becoming involved in research during medical school. This will be achieved using a selection of Likert scale, multiple choice and free text questions Ordinal logistic regression analysis will be performed to understand the association between specific factors and student involvement in research. This study will also characterise the proportion of medical students who are currently interested in conducting research in the future.

Ethics and dissemination

Ethics approval has been obtained from the Medical Sciences Interdivisional Research Ethics Committee, Oxford, England. The results will be disseminated via publication in a peer-reviewed medical journal and may be presented at local, regional, national, and international conferences by medical student collaborators.

Strengths and Limitations of this Study

- We will be the first to examine the attitudes of current UK medical students towards previous and future research opportunities
- The design is limited by its snapshot nature, as we are unable to see how attitudes have or will change over time
- It is possible that those already interested in this area will be more likely to complete the survey

Introduction

It is of vital importance to the progress of medical innovation for clinicians to perform research as their combined academic and clinical perspectives allow the most pressing challenges in health care to be identified and studied. Their position affords an integrative outlook, evaluating practice objectively while advancing our collective knowledge [1]. Clinicians playing an active role in research often ensures that advances made in that research are incorporated into clinical practice as efficiently as possible [2,3,4].

While it is difficult to quantify the total number of clinicians involved in research, the total number of clinical academic staff employed by UK medical schools has declined since its peak in 2010 [5]. An insufficient number of individuals entering the clinical academic 'pipeline' has been identified as a contributing factor in this downward trend [5], resulting in reduced replacement of an aging workforce. This is surprising given that involvement in research is often seen as a desirable quality in the CVs of medical students and junior doctors [6]. Indeed, one survey found career success was independently associated with having conducted research as a student [7] . Furthermore it has been shown that intercalation can have positive academic consequences [8] . Additionally, it is a GMC requirement for students to have a working knowledge of research [9]. It is therefore important to gauge the aspirations and career goals of students, as well as their attitudes towards careers which involve research and clinical academia.

While there have been attempts to survey the barriers and enablers of progression for early career clinical academics [10,11] there are currently no published systematic surveys of medical students which seek their views on doing research both as a student and during their career as a doctor. Furthermore, recent changes to the centralised application system has meant that research publications and additional degrees are no longer granted points as of 2023 [12,13], despite such points having been shown to be one of the primary motivating factors for medical student involvement in research [14,15]. This change has proved controversial [13,15] and several authors have raised concerns that fewer medical students will now become involved with research.

Given the need to maintain, and indeed increase, the number of clinicians involved in research, it is important to identify the factors influencing medical students in deciding whether to engage in research. Previous studies have identified intrinsic demographic factors which may affect interest in particular specialty training programmes, including academic training pathways, and research, such as

For peer review only

economic background, ethnicity, and gender [16,17]. Non-demographic influences have also been identified, including previous involvement in research and access to mentors [16,17,18]. Similar data is however currently lacking for medical schools in the UK, and no clear data available for the interactions between demographic and non-demographic influences. With variation in both medical school requirements for involvement in research during training and opportunities to intercalate [19], it is important that we understand the different attitudes towards clinical academia amongst UK medical students. We also hope to describe the experiences that have conceivably shaped these, and the perceived barriers to pursuing research during medical school and beyond.

The Survey of Medical student Attitudes to Research and Training pathways (SMART) study is an online national questionnaire-based study. The aim of this study is to ascertain medical student perceptions of research and research-orientated careers, including perceived barriers and incentives to participating in research as a student and in following a research-orientated career. It is our hope that increasing awareness of the issues medical students face will encourage solutions to be sought by medical schools and regulators. SMART will also identify key issues for the Academy of Medical Sciences to address as part of their long-term INSPIRE strategy [20].

Methods and Analysis

Primary aim:

- To determine current medical students' involvement with research

Secondary Aims:

- To characterise the group of students who are interested in research
- To identify the factors that drive medical students to conduct research
- To identify the perceived barriers that have historically and continue to prevent current medical students from partaking in research
- To determine if current medical students are interested in conducting research
- To identify factors that could encourage current medical students to conduct research

Study Design

SMART is an online, national, multi-centre, questionnaire-based study focussing on medical student perceptions of research and research-orientated careers, including barriers and incentives to participating in research as a student and in following a research-orientated career. The questionnaire will be disseminated through collaborative university medical school and student networks, such as the network of INSPIRE leads across the country. It is well documented that the generic collaborative method works, and that participating students benefit from involvement [21,22,23,24,25,26]. In short the collaborative method involves a 'snap-shot, protocol-driven, pragmatic multicentre research' (National Research Collaborative and Association of Surgeons in Training Collaborative Consensus Group 2018

p355), approach undertaken by separate groups of trainees, or in this case students; the collaborative method also allows for greater size and power of studies [21]. The SMART study will be delivered by a team of University of Oxford medical students in clinical years 4, 5 and 6. The questions included can be found in appendix S1. The questions were formulated by medical students at Oxford University. A brief review of the existing literature was performed to identify the gaps in knowledge and to also look at similar questionnaires and qualitative studies on the viewpoints of students and academics. This allowed an understanding of domains and items relevant to determining the aim of the project. Two students separately suggested questions which were pooled before being considered by all authors. Medical student and academic staff feedback was also sought at this point. The questionnaire has face and content validity, there is no gold standard to compare against for criterion validity. Questions were adjusted following reviewer comments to make them as non-directive as possible. Construct validity will be checked during the data collection period. Feedback will be collected from a pilot study regarding the suitability of questions, length of questionnaire and suggestions for other aspects to consider.

Patient and public involvement

No patients or members of the public were involved in the design of the study. We will share results with interested individuals and publicly via journal publication.

Methods for recruiting participants

All medical students currently studying for a UK medical degree at a UK medical school recognised by the General Medical Council (GMC) will be eligible to participate. A list of these medical schools can be found in Appendix S2.

Medical students will be invited to participate in the study through several routes:

- Medical Societies
- Medical School mailing lists
- Organisations focused on academic medicine, such as the Academy of Medical Sciences
- Conferences
- Freshers' Fairs
- Social Media

In addition, medical students enrolled in the medical schools in Appendix S2 will be invited to collaborate in the study as regional leads as described in Table 1. The maximum number of collaborators from each medical school will be one per year. Appendix S3 details the participant facing information used in recruitment for the SMART study.

Collaborators will ensure that their medical school is formally engaged at an early stage of this study, and they will be primarily responsible for disseminating this questionnaire amongst students at their medical school. Medical school collaborators

For peer review only

will be able to request for their own specific data and the analysis done on said data from the SMART steering committee following study completion. This data will be anonymised. Researchers will have access to this anonymous data.

Dates	Activity
**1st September 2021 – 1st October 2021**	Study pilot to be run at Oxford
**1st April 2021 – 30th May 2021**	Recruit collaborators at all UK universities via collaboration with INSPIRE Student Leads
**1st October 2021 – 30th November 2021**	Study to be modified based on pilot feedback
**1st September 2021 – 1st October 2021**	Study set up e.g. training collaborators, gaining approval for this study at each centre, providing collaborators with Qualtrics logins
**1st November 2021 – 1st January 2022**	Study runs nationally for 2 months
**1st January 2022 – 31st March 2022**	Analyse data and prepare manuscript

Table 1: Project timeline. Extended data collection periods may be incorporated to grant flexibility to centres that may have experienced logistical obstacles to study commencement.

Information provided to participants

The following pieces of information will be provided to participants before taking part in the study. It will be attached to recruitment emails, and appear as the front page of the questionnaire:

- Name of the study: Cross-Sectional Survey of Medical student Attitudes to Research and Training pathways (SMART) study
- Name of the principal researcher carrying out the study and information on how to contact him: Soham Bandyopadhyay, soham.bandyopadhyay@st-hildas.ox.ac.uk
- **What is the aim of this study?** This study aims to ascertain current medical student involvement with research. We also hope to identify factors encouraging and discouraging students from partaking in research and to consider what may encourage more engagement with scientific research in the future.
- **Why have I been selected to take part?** You are being invited to take part in the questionnaire as you are a medical student currently studying for a UK medical degree at a UK medical school recognised by the General Medical Council (GMC).
- **What do I have to do?** If you choose to participate in this voluntary survey, you will be asked to complete a questionnaire about your background, your previous exposure to research and your feelings towards a research career. This study is voluntary. If you decide not to participate this will not impact your academic standing in any way. If you decide to take part, you will be asked to

For peer review only

complete the survey by clicking on the link below. This survey is expected to take about 10 -15 minutes to complete, but there is no time limit and you can take as much time as you like. No background knowledge is required. We will ask for your consent for the collection and storage of data in accordance with the UK General Data Protection Regulation (GDPR) within the survey. For more information on GDPR please click on the following link: https://gdpr-info.eu/.

- **Do I have to participate?** Please note that your participation is voluntary. You may withdraw at any point during the questionnaire for any reason, before submitting your answers, by closing the browser. In cases of withdrawal from the study, no new data will be collected or linked to other data from that point on. If you do not want to answer some of the questions you do not have to, but you can still be in the study. All questions are optional. Your decision whether or not to be part of the study will not affect your academic standing or your access to university support services. If you have already submitted data and wish to withdraw from the study, please contact soham.bandyopadhyay@st-hildas.ox.ac.uk by 1st October 2021.
- **Who has approved this study?** This project has received ethics clearance through the University of Oxford's ethical approval process for research involving human participants, reference R73479/RE001.
- **How will my data be used?** Your answers will be completely anonymous, and we will take all reasonable measures to ensure that they remain confidential. Your data will be stored in a password-protected file and may be used in academic publications. Your IP address will not be stored. If you provide us with your email address, we will delete that information at the end of the study. No answers will be linked to your email address. Research data – your anonymised answers – will be stored for a minimum of ten years after publication or public release.
- **Who will have access to my data?** Qualtrics is the data controller with respect to the personal data they hold about you and, as such, will determine how your personal data is used. Please see their privacy notice here: https://www.qualtrics.com/privacy-statement. Qualtrics will share any email address you provide and your anonymised answers with the University of Oxford, for the purposes of research. Researchers involved in the project will have access to this anonymised data. The University of Oxford is the data controller of university email addresses, please see their privacy notice here: https://compliance.admin.ox.ac.uk/student-privacy-policy. Responsible members of the University of Oxford and funders may be given access to data for monitoring and/or audit of the study to ensure we are complying with guidelines, or as otherwise required by law.
- **Are there any benefits to taking part?** Despite not have any immediate individual benefits by participating in this survey, you are given the opportunity to contribute to valuable and innovative research which could be used in the future by medical universities and the world. You may find this survey an opportunity to self-reflect. There will be the option to submit email address in order to be entered into a prize draw. At the conclusion of data collection, two random participants will be awarded £50 in Amazon vouchers, two further random participants will be awarded £25 in Amazon vouchers. This will be optional, as it requires you to provide personally identifying data (i.e. contact

For peer review only

details). These will not be linked to the questionnaire answers given, and will only be used for contact regarding relevant rewards as above.

- **Will the research be published?** The findings of the study may be published in peer reviewed journals, presented at relevant conferences and meetings and a summary of the findings will be made available on social media**.**
- **Are there any possible risks involved with my participation?** Some of the questions that we ask may cause upset. If you experience any distress from participating in this study, you may stop the survey at any time or skip any upsetting questions. If your distress continues after leaving the survey, we have provided a list of supportive services nationwide that can be helpful and that you might consider contacting (appendix S4, to be linked here, and appear again at the close of the survey).
- **Who do I contact if I have a concern about the study or I wish to complain?** If you have a concern about any aspect of this project, please speak to the researcher Soham Bandyopadhyay at soham.bandyopadhyay@st-hildas.ox.ac.uk who will do her best to answer your query. The researchers should acknowledge your concern within 10 working days and give you an indication of how they intend to deal with it. If you remain unhappy or wish to make a formal complaint, please contact the Chair of the Medical Sciences Inter-Divisional Research Ethics Committee: Email: ethics@medsci.ox.ac.uk; Address: Research Services, University of Oxford, Wellington Square, Oxford OX1 2JD OR The Chair will seek to resolve the matter in a reasonably expeditious manner.
- **How do I find out what was learned in this study?** This study is expected to be completed by approximately March 2022. If you would like a brief summary of the results, please write to us by email to request information
- **Who to contact for further details?** For any further questions or more information on the study, please contact us on the following email address: soham.bandyopadhyay@st-hildas.ox.ac.uk. Alternatively, you could contact principal investigator Dr Catherine Swales at catherine.swales@ndorms.ox.ac.uk.

A representation of how this information will be presented to medical students can be found in Appendix S5.

Financial and other rewards to participants

A prize draw involving participants who have opted to provide their contact details will take place at the conclusion of data collection. Two random participants will be awarded £50 in Amazon vouchers. Two further random participants will be awarded £25 in Amazon vouchers..

Data Collection

The online questionnaire consists of 23 quantitative and qualitative questions that utilise a combination of the Likert scale, multiple choice options and free text in order to broaden the capture of sentiment nuance and improve precision in the data. The questionnaire has been sent to medical students who were not involved in creating the data collection proforma. Their responses were evaluated for potential problems,

For peer review only

and the questionnaire was updated to ensure the questions were relevant, comprehensive and accessible. A pilot study will be performed at Oxford Medical School before this study is launched nationally. There are 952 students enrolled at Oxford Medical School. Data collection will take place at the beginning of September 2021, aiming to be complete by November 2021. Based on the experiences from the pilot, the questionnaire will be modified to improve clarity, objectivity, and acceptability (appendix S6).

Primary Outcome

To describe the extent of current medical students' involvement with research

Secondary Outcomes

The identify and understand the reasons driving and excluding medical students from research. To identify factors that might encourage more current students to conduct research.

Data management

All the data will be anonymised and stored in Qualtrics. Qualtrics is cloud-based platform, with the ability to create and customise databases. Qualtrics guarantees the highest levels of security for stored data and is compliant with regulations including GDPR and ICH E6 Good Clinical Practice [27]. The research team will have individual accounts, with access to data determined by the study administrator. Audit logs are also available to provide an overview of data access and modifications.

Statistical Analysis

We will use descriptive statistics to achieve our primary outcome in describing the extent of current medical students' involvement with research.

In order to achieve our secondary outcomes we will consider the following research questions using machine learning techniques to predict in our cohort:
Q1: What factors are associated with whether an individual in our cohort intends to pursue an academic career?

Q2: What factors are associated with a respondent's interest in undertaking (more) research in the future?"

Statistical procedure:

Unless explicitly stated otherwise, assume alpha=0.05, and CI = 95%. Data will be cleaned of any conflicting, impossible, or corrupted data by deletion of all entries of the same individual.

1. Data processing for descriptive statistics:

This section seeks to provide an easy to interact with description of the responses to our survey, and the demographics of those who responded.

1. Percentages for responses by each question.
2. Median and deciles of spread for answers to appropriate question(s).
3. Mode for answers to appropriate question(s).

For peer review only

1. Number of missing values per question. Data will be assumed to be missing completely at random.
4. Number of individuals with 1 or more missing values.
5. Identify sampling weights for demographic populations utilizing data from local sources, and national data collected about medical student demographics at each school.
6. Key qualitative themes identified with free writing texts of questions 15, 16, 17, 23. We will look to identify common sentiments, having trained multiple different team members to independently look across the data and identify recurrent themes and sentiments. Each response will be evaluated by at least two of the trial team.

2. Establishing correlation across responses.

The tests described in this section aim to find any predictive associations and identify likely explanatory variables. As a method, it also allows capture of the entirety of the cohort data.

We will perform Multiple Correspondence Analysis, utilizing sampling weights from 1.5. We will account for ordinality in data with orthogonal polynomials [28], where appropriate, to produce a simple visualisation of correlation across variables. All interval/ratio variables will be appropriately binned to produce ordinal variables for the analysis above. If there are a low number of responses then answers to question 4 may be binned into broader ethnicities and backgrounds, in order to maintain statistical power.

3. Testing Q1: What factors are associated with whether an individual in our cohort intends to pursue an academic career?

We will run an ordinal logistic regression, using the cumulative logit link function. We will use the ordinal responses to question 20. ‘How much do you agree with the statement: “I wish to pursue an academic career or an academic training pathway.” ’ (Likert scale of “Disagree” through to “Strongly agree”.) ”), as our response variable, and use as predictor variables the answers to other questions. Detail on proposed model construction can be found in appendix S7.

4. Q2: What factors are associated with a respondent's interest in undertaking (more) research in the future?"

The methods will be the same as above, using the ordinal responses to question 22. How strongly do you agree with the statement: “I would be interested in undertaking (more) research in the future.” (Likert scale of “Disagree” through to “Strongly agree”.) as our response variable, with answers to all questions as predictor variables as detailed in appendix S8 model construction.

5 Subgroup analysis.
With a priori assumptions about significant contributors to variance, one may attempt to control for these through subgroup analysis, in categorisation of the data points by a given variable or variables.

However, to ensure validity of causal inference, one must ensure that the appropriate covariates are selected for any regression model attempting to

For peer review only

interrogate such data [29]. We believe a priori that we may see a large degree of variance between those who have already completed research, and those who have not. To account for this, we propose two models (appendix S9, equations 3 and 4) to account for this difference and ascertain key contributors in these different groups, with the goal of more policy-relevant inferences being drawn for each subgroup.

Additional subgroup analysis may be carried out if key contributors to variance are identified in the Multiple Correspondence Analysis, or otherwise. Additional exploratory analysis may be carried out as deemed necessary, but it will be reported as such, and will be carried out with good statistical reporting in mind.

Authorship

In accordance with National Research Collaborative (NRC) authorship guidelines [24], all publication outputs from SMART will be listed under a unified corporate authorship: ‘SMART Collaborative’. Certain publications will include named authors on the bye-line as well as the group name. This will follow the example set by commendable collaboratives including STARSurg and InciSioN UK [25,26]. Anyone who has demonstrated satisfactory completion of the minimum requirements for authorship will be eligible for PubMed-citable collaborative authorship in accordance with the roles defined below:

Writing Group: Responsible for the overall scientific content, data analysis, and preparation of research manuscripts.

Steering Committee: Responsible for the protocol design, project coordination, and data handling.

Collaborators: A network of medical students across all medical schools. They are responsible for leading the study regionally.

Ethics and dissemination

Ethics

Ethics approval has been obtained from Medical Sciences Interdivisional Research Ethics Committee, Oxford, England. Reference R73479/RE001.

Dissemination

The protocol will be disseminated primarily through recruited medical student collaborators. Should UK medical schools wish to see the protocol, collaborators may pass it along as well. Any publications of the protocol will be advertised through social media.

Following study completion, teleconferences will be held with all collaborators to share and discuss the data analysis undertaken and the study results. Following this, the results will be presented at local, regional, national, and international conferences by medical student collaborators. A standard PowerPoint presentation and poster will be created for this purpose. All presentations will be coordinated by

For peer review only

the SMART steering committee to avoid duplications and to ensure all conference regulations are fulfilled. In addition, the results will be disseminated via publication in a peer-reviewed medical journal. All collaborators will be given PubMed citable collaborative co-authorship under the institutional name 'SMART Collaborative'. We will have a hybrid authorship list of named authors and the institutional collaborative.

Following publication, the manuscript can be shared by collaborators with their medical schools to feedback the study results, and to highlight the scope for expanded integration of research within medical school curricula. Medical schools can request for their own specific data and the analysis done on said data from the steering committee following study completion. The fully anonymised dataset will be made publicly available.

Word count: 3999

References

1. Jacob H (2016) Why all doctors should be involved in research. BMJ 352:i164

2. Rees, M., Dangerfield, P., Katz, D., & Schofield, M. Every Doctor a Scientist and a Scholar. *BMA Publications 2015*.

3. BMA. The Role of the Clinical Academic. *BMA Publications 2014.*

4. Savill J. More in expectation than in hope: a new attitude to training in clinical academic medicine. *BMJ 2000*; 320:630

5. Medical Schools Council. Survey of medical clinical academic staffing levels 2017. www.medschools.ac.uk/SiteCollectionDocuments/Survey-Medical-Clinical-Academic-Staffing-Levels-2017.pdf.

6. Chambler AF, Chapman-Sheath PJ, Pearse MF. A model curriculum vitae: what are the trainers looking for*? Hospital Medicine 1998*;59:324–26.

7. Brancati FL, Mead LA, Levine DM et al. Early predictors of career achievement in academic medicine. *JAMA. 1992*;267(10):1372-6.

8. Mahesan N, Crichton S, Sewell H, Howell S. The effect of an intercalated BSc on subsequent academic performance. *BMC Med Educ*. 2011 Oct 3;11:76.

9. General Medical Council. Outcomes for Graduates. [online] Available at: https://www.gmc-uk.org/education/standards-guidance-and-curricula/standards-and-outcomes/outcomes-for-graduates/outcomes-for-graduates (Accessed May 2021)

10. Lopes J, Ranieri V, Lambert T*, et al.* The clinical academic workforce of the future: a cross-sectional study of factors influencing career decision-making among clinical PhD students at two research-intensive UK universities. *BMJ Open* 2017; **7:**e016823.

11. Oldfield K, Turner C, Garnett E. A Cross-Funder Review of Early-Career Clinical Academics: Enablers and Barriers to Progression. Available at <https://mrc.ukri.org/documents/pdf/review-of-early-career-clinical-academics/> [Accessed 12 May 2021].

12. *MSC Statement on the UKFPO decision to remove Educational Achievements from the Foundation ranking process | Medical Schools Council*. [online] Available at: <https://www.medschools.ac.uk/news/msc-statement-on-the-ukfpo-decision-to-remove-educational-achievements-from-the-foundation-ranking-process> [Accessed 12 May 2021].

13. British Medical Association. *Additional achievements ruled out of foundation programme applications*. [online] Available at: <https://www.bma.org.uk/news-and-opinion/additional-achievements-ruled-out-of-foundation-programme-applications> [Accessed 12 May 2021].

14. Pang KH, Hobbis C, Burleigh EJ, Miah S. Publication Component of the UK Foundation Programme Application: Perception of Medical Students. *Adv Med Educ Prac*t. 2020 Oct 8;11:735-740.

15. McVeigh J, Jeilani M, Super J. Factors influencing the engagement in research of medical undergraduates and clinical trainees. *Postgraduate Medical Journal* Published Online First: 16 February 2021.

16. Mina S, Mostafa S, Albarqawi HT et al. Perceived influential factors towards participation in undergraduate research activities among medical students at Alfaisal University-College of Medicine: A Saudi Arabian perspective. *Med Teach. 2016*;38 :S31-6

17. Amgad M, Man Kin Tsui M, Liptrott SJ, Shash E. Medical Student Research: An Integrated Mixed-Methods Systematic Review and Meta-Analysis. PLoS One. 2015 Jun 18;10(6):e0127470.

18. Ejaz K, Shamim MS, Shamim MS, et al. Involvement of medical students and fresh medical graduates of Karachi, Pakistan in research. *J Pak Med Assoc. 2011*;61(2):115-20.

19. Nicholson JA, Cleland J, Lemon J. Why medical students choose not to carry out an intercalated BSc: a questionnaire study. *BMC Med Educ 2010*;10:25-6920-10-25.

20. Acmedsci.ac.uk. 2021. *INSPIRE | The Academy of Medical Sciences*. [online] Available at: <https://acmedsci.ac.uk/grants-and-schemes/mentoring-and-other-schemes/INSPIRE> [Accessed 12 May 2021].

21. National Research Collaborative & Association of Surgeons in Training Collaborative Consensus Group. Recognising contributions to work in research collaboratives: Guidelines for standardising reporting of authorship in collaborative research. *Int J Surg. 2018*;52:355-60.

22. Bhangu A, Fitzgerald JE, Kolias AG. Trainee-led research collaboratives: a novel model for delivering multi-centre studies. *ANZ J Surg. 2014*;84(12):902-3.

23. Chapman SJ, Glasbey JC, Khatri C, et al. Promoting research and audit at medical school: evaluating the educational impact of participation in a student-led national collaborative study. *BMC Med Educ. 2015*;15(1):47.

24. National Research Collaborative & Association of Surgeons in Training Collaborative Consensus Group. Recognising contributions to work in research collaboratives: Guidelines for standardising reporting of authorship in collaborative research. *Int J Surg. 2018*;52:355-60.

25. STARSurg Collaborative. Prognostic model to predict postoperative acute kidney injury in patients undergoing major gastrointestinal surgery based on a national prospective observational cohort study. *BJS Open. 2018*;2(6):400-10.

26. Bandyopadhyay S, Shortland T, Wadanamby SW, Thomas HS, Gurung B, Akhbari M, et al. Global Health Education in Medical Schools (GHEMS) study protocol. *J Glob Health Rep.* 2019; 3:e2019052.

27. European Medicines Agency. Available at <https://www.ema.europa.eu/en/documents/scientific-guideline/ich-e-6-r2-guideline-good-clinical-practice-step-5_en.pdf>. [Accessed 12 May 2021].

28. Rosaria Lombardo & Eric J. Beh (2010) Simple and multiple correspondence analysis for ordinal-scale variables using orthogonal polynomials, *Journal of Applied Statistics*, 37:12, 2101-2116, DOI: 10.1080/02664760903247692

29. Johannes Textor, Benito van der Zander, Mark S Gilthorpe, Maciej Liśkiewicz, George TH Ellison, Robust causal inference using directed acyclic graphs: the R package 'dagitty', *International Journal of Epidemiology*, Volume 45, Issue 6, December 2016, Pages 1887–1894

Authors' contributions

The study concept and design was conceived by SB and SR. Abstract was written by GM, PI and SR. Strengths and limitations were written by SR. Introduction was written by SR, RB and HE. Methods were written by SR. Statistical analysis was written by SR and AG-R. AG-R provided the statistical planning, analysis, and contributed to survey design. . Authorship was written by SR. Ethics and dissemination was written by SR. CS and SB had a supervisory role at all stages of manuscript preparation, helped prepare and write funding applications and critically reviewed the manuscript. All authors critically reviewed the manuscript.

Funding

This work was supported by an INSPIRE grant from the Wellcome Trust [20]. No grant number is available.

Competing interests

None declared

For peer review only

Appendix S1

Proforma of questionnaire, to be converted to digital format for administration. Text in red describes the available answers for each question.

Q1a. Which medical school do you attend? (If currently intercalating at a separate university, please give the university from which you will receive your main degree).

☐ Please select your university from the dropdown list *as in appendix S2*

Q1b. Which year of medical school are you currently in? (Ordinal)

☐ Please select which year of your medical degree you are currently enrolled in (First/Second/Third/ /Year 4 of 5/ Year 4 of 6/Year 5 of 6/ Year 5 of 5 /Year 6 of 6/ Intercalation/ Graduate Entry first/ Graduate Entry second/ Graduate Entry third/ Graduate Entry fourth

Q2. Have you already completed an academic degree (Bachelor's/Masters/Doctorate)? (binary)

☐ Please select whether you currently already have a completed higher education degree, such as a Bachelor's, Master's, or Doctorate. (YES/NO)

Q3. If you answered yes to Question 2, please select all those degrees you currently have. (Nominal/Ordinal)

☐ Please select all degrees you currently have. (Bachelor's – in a scientific degree/Bachelor's – in an arts degree/Master's – in a scientific degree/Master's – in an arts degree/Doctorate/ Other (please type))

Q4. Choose one option that best describes your ethnic group or background? (Nominal)

☐ Please select which ethnic group you most strongly identify as. (English, Welsh, Scottish, Northern Irish, British/Irish/Gypsy or Irish Traveller/Any other White background/ White and Black Carribean/ White and Black African/ White and Asian/ Any other Mixed or Multiple ethnic background/ Indian/ Pakistani/ Bangladeshi/ Chinese/ Any other Asian background/ African/Caribbean/ Any other Black, African, or Caribbean background/Arab/ Any other ethnic group)

*Note, list taken from government list of ethnic groups https://www.ethnicity-facts-figures.service.gov.uk/style-guide/ethnic-groups

Q5. What best describes your gender (Nominal)

☐ Female/ Male/ Prefer not to say/ Prefer to self-describe (free text)

Q6. Do you identify as LGBTQ+? (Binary)

☐ Please select whether you identify as LGBTQ+ (Lesbian, Gay, Bisexual, Transgender, Queer/Questioning, or any other part of the LGBTQ+ community). (YES/NO)

Q7. During school, at any point, were you eligible for free school meals? (binary)

☐ Please select whether you were, at any point, eligible for free school meals during school. (Yes/No)

Q8. How many of your first degree relatives are or have been a healthcare professional*? (Ratio/Ordinal)

☐ Please select the number of relatives who currently or have ever identify/identified as healthcare professionals. (0,1,2,3,4,5,6,7,8,9,10,10+)

*Healthcare professional defined according to WHO international classification of healthcare workers as professionals who study, advise on or provide preventive, curative, rehabilitative and promotional health services based on an extensive body of theoretical and factual knowledge in diagnosis and treatment of disease and other health problems. This includes:

- General medical practitioners
- Specialist medical practitioners
- Nursing professionals
- Midwifery professionals
- Traditional and complementary medicine professionals
- Paramedical practitioners
- Dentists
- Pharmacists
- Environmental and occupational health and hygiene professionals
- Physiotherapists

For peer review only

- Dieticians and nutritionists
- Optometrists and ophthalmic opticians

Q8b. If you answered >0 Question 8, how many of your relatives are or have been held an academic position* in the healthcare environment? (Ratio/ordinal)

☐ Please select all the number of relatives who currently or have ever identify/identified as academics in the healthcare environment. (0,1,2,3,4,5,6,7,8,9,10,10+)

*Academic position defined as working within a research or teaching capacity within a higher education institute. This includes, but is not limited, to, <u>professors, readers, senior lecturers, lecturers, post-doctoral researchers, research fellows, teaching fellows</u>

Q9. How many of your first degree relatives are or have been in academia? (Ratio/ordinal)

☐ Please select the number of relatives who currently or have ever identify/identified as being in academia. (0,1,2,3,4,5,6,7,8,9,10,10+)

Q9b. If you answered >0 Question 9, how many of your relatives are or have been held an academic position in the healthcare environment? (Ratio/ordinal)

☐ Please select all the number of relatives who currently or have ever identify/identified as academics in the healthcare environment. (0,1,2,3,4,5,6,7,8,9,10,10+)

*Academic position defined as working within a research or teaching capacity within a higher education institute. This includes, but is not limited, to, <u>professors, readers, senior lecturers, lecturers, post-doctoral researchers, research fellows, teaching fellows</u>

Q11. In which area did you undertake the majority of your pre-university education?

☐ Please select the geographical area where you undertook the majority of your education before university (UK, EU, outside EU)

For peer review only

Q12. How well do you feel your medical school has educated you about research? (Ordinal)

☐ Please select which best represents your opinion. (Not well at all/ Less than adequately/Adequately/More than adequately/Very well)

Q13. How much research have you undertaken to date? (Ordinal)

☐ Please select how much research you feel you have undertaken to date. (None/A little bit/ A fair amount/Quite a lot/A very significant body of research)

Q14. If you have undertaken any research, was any of this a compulsory part of your degree? (Binary)

☐ Please select whether any research you have done included a compulsory part of your degree. (Yes/No)

Q15. If you have not yet been involved in voluntary research, what have been the barriers preventing this? (Nominal)

☐ Please describe the barriers that have prevented you from undertaking research outside of any compulsory degree obligation. (Free text up to 500 words)

Q16. Please tick any of the following that apply to your research. You may tick more than one box or none at all : (Nominal)

☐ Please tick any of the following that apply to your research: (QIP/audit/basic science project/ clinical project/ co-author on original paper in peer reviewed journal/ co-author on any publications related to research/ named collaborator on original paper in peer reviewed journal/ named collaborator on any publication related to research/ presented a poster/ given an oral presentation/other (please type))

For peer review only

Q17. Why did/do you do research? (Nominal)

☐ Please tick any of the following that apply to your research:. (Interest in scientific problems/Interest in the subject/Personal development/Extra income/contribution to better health care/Improving critical thinking/Career progression/Intellectual stimulation/Feel obliged to do it/Other (please type)

Q18. How much do you think research is useful in combination with your medical studies? (Ordinal)

☐ Please select to what degree you feel research is useful in combination with your medical studies. (Not at all useful/ Somewhat useful/ A little useful/A lot useful/Very useful)

Q19. How difficult is it to combine research with your medical studies? (Ordinal)

☐ Please select how difficult you find it to integrate research with your medical studies. (Not at all difficult/Somewhat difficult/A little difficult/ A lot difficult/ Very difficult)

Q20. How much do you agree with the statement: "I wish to pursue an academic career ." (Ordinal)

☐ Please select how strongly you intend to pursue an academic career. (Strongly disagree/ Somewhat disagree/No opinion/Somewhat agree/Strongly agree)

Q21. How much do you agree with the statement: "I wish to pursue an academic training pathway ." (Ordinal)

Please select how strongly you intend to pursue an academic career. (Strongly disagree/ Somewhat disagree/No opinion/Somewhat agree/Strongly agree)

Q22. How much do you agree with the statement: "I would be interested in undertaking (more) research in the future." (Ordinal)

☐ Please select how strongly you agree with the above statement. (Strongly disagree/ Somewhat disagree/No opinion/Somewhat agree/Strongly agree)

Q23. What would encourage your involvement in research in the future? (Ordinal)

☐ Please tick all that apply (More time/More incentives/ Easier access to research groups and projects/ Clearer information about how to get involved/ Clearer information about benefits of research/ Guaranteed rewards e.g. publication or presentation/ Other (please type)

Appendix S2

A list of medical schools recognised by the GMC as of 1st January 2020:

The University of Aberdeen
The University of Birmingham
The University of Bristol
The University of Buckingham
The University of Cambridge
Cardiff University
The University of Dundee
The University of East Anglia
The University of Edinburgh
University of Exeter Medical School
The University of Glasgow
The Imperial College of Science, Technology and Medicine
Keele University
King's College London
Lancaster University
The University of Leeds
The University of Leicester
The University of Liverpool
The University of London
The University of Manchester
The University of Newcastle
The University of Nottingham
The University of Oxford
Plymouth University Peninsula Schools of Medicine and Dentistry
Queen Mary University of London
The Queen's University of Belfast
St George's Hospital Medical School
Swansea University
The University of Sheffield
The University of Southampton
University College London
The University of Warwick
A combination of the University of Brighton and the University of Sussex
A combination of the University of Hull and the University of York

For peer review only

Appendix S3

This document outlines the participant facing information used in recruitment for the SMART study

1. **Enrolment and 1st Survey Phase:** Link shared via email and social media
    a. Initiation: 1st – 14th November
2. **1st and 2nd Follow up for 1st Survey**
    a. 14th November – 30th November
3. **3rd Follow up for 1st Survey**
    a. 31st November – 1st January

1. Initial email to students

Dear Students,

We are a group of researchers from across the country who are interested in understanding the medical student perceptions of research and research-orientated careers.

We invite you to participate in a 15-minute voluntary survey that is collecting information on your views on research and research-orientated careers. When you access the survey, you are going to be asked a variety of questions about your background, your previous experiences of research, and your thoughts on a future research career. Your IP address will not be recorded. All answers will remain anonymous and confidential.

If you wish to partake in our prize draw, please provide your email address when requested by the survey. This email address will not be linked to your answers. More information on the risks and benefits of completing this survey are included in the survey link. **You can access the survey here:**

<INS LINK>

If you have any questions, you can contact a member of the research team by email at soham.bandyopadhyay@st-hildas.ox.ac.uk
Thank you for your consideration.

Many thanks,
<Your name>
SMART team

2. First and Second Follow-up email for 1st survey

Dear Students,

Just a friendly reminder that the SMART survey will remain open until January 1st, 2022. Please take a few moments to participate in this survey if you have not done so yet.

For peer review only

We invite you to participate in a 15-minute voluntary survey that is collecting information on your views on research and research-orientated careers. When you access the survey, you are going to be asked a variety of questions about your background, your previous experiences of research, and your thoughts on a future research career. Your IP address will not be recorded. All answers will remain anonymous and confidential.

If you wish to partake in our prize draw, please provide your email address when requested by the survey. This email address will not be linked to your answers. More information on the risks and benefits of completing this survey are included in the survey link. **You can access the survey here:**

<INS LINK>
If you have any questions, you can contact a member of the research team by email at soham.bandyopadhyay@st-hildas.ox.ac.uk

Thank you for your consideration.

Many thanks,
<Your name>
SMART team

3. Third Follow-up email for 1st survey (sent out the day before the survey closes)

Dear Students,
Just a friendly reminder that the SMART survey will remain open until January 1st, 2022. Please take a few moments to participate in this survey if you have not done so yet.

We invite you to participate in a 15-minute voluntary survey that is collecting information on your views on research and research-orientated careers. When you access the survey, you are going to be asked a variety of questions about your background, your previous experiences of research, and your thoughts on a future research career. Your IP address will not be recorded. All answers will remain anonymous and confidential.

If you wish to partake in our prize draw, please provide your email address when requested by the survey. This email address will not be linked to your answers. More information on the risks and benefits of completing this survey are included in the survey link. **You can access the survey here:**

<INS LINK>
If you have any questions, you can contact a member of the research team by email at soham.bandyopadhyay@st-hildas.ox.ac.uk

Thank you for your consideration.

Many thanks,
<Your name>

For peer review only

SMART team

Appendix S4

Thank you for taking part in this research study. This information is intended for anyone who may be concerned or distressed after participating in this study. If this applies to you, I would like to point out that there are several sources of advice or help which are free and readily available to you and which may prove useful. Specifically, these include (please note, you may need to copy and paste the web addresses into your browser):

Your GP: Book an appointment with your GP. They can offer advice or refer you to other more specific services to get help.

Your University Counselling Service (where available)

NHS 111

FRANK: For friendly, confidential drugs advice, call FRANK on: 0300 123 6600 (0800776600 in Scotland) . Or, you can access their website at: http://www.talktofrank.com

MIND: Mind is a mental health charity serving England and Wales. To ask about mental health or mental health services, or to find out more about a particular Mind service, phone the MIND Information Line on 0300 123 3393. Trained staff and volunteers will be able to give you details of services in your area and talk through options with you.
https://www.mind.org.uk/

Students Against Depression: An award-winning website offering information, blogs and resources to help students find their way forward from low mood or depression: http://www.studentsagainstdepression.org

Student Minds: Student Minds is a national student mental health charity working to encourage peer interventions for student mental health. For more information, take a look at their website at: http://www.studentminds.org.uk

The Samaritans: Trained volunteers are able to listen to you any time day or night. We can help you talk through whatever is troubling you, find the answers that are right for you, and offer support. Call on: 116 123
https://www.samaritans.org/

LGBT foundation: a national charity delivering advice, support and information services to lesbian, gay, bisexual and trans (LGBT) communities
https://lgbt.foundation/

IN AN EMERGENCY: If you are experiencing suicidal thoughts and think that you might be unable to keep yourself safe, visit your nearest A&E department or call 999.

For peer review only

Appendix S5

Survey of Medical student Attitudes to Research and Training pathways (SMART) study

Thank you for your interest in participating in this study. Please take a moment to read the following information, before ticking the box to confirm your participation in the study.

If you have any questions or concerns, please contact the principal researcher, Soham Bandhyopadhyay, at soham.bandyopadhyay@st-hildas.ox.ac.uk

- **What is the aim of this study?** This study aims to ascertain current medical student involvement with research. We also hope to identify factors encouraging and discouraging students from partaking in research and to consider what may encourage more engagement with scientific research in the future.
- **Why have I been selected to take part?** You are being invited to take part in the questionnaire as you are a medical student currently studying for a UK medical degree at a UK medical school recognised by the General Medical Council (GMC).
- **What do I have to do?** If you choose to participate in this voluntary survey, you will be asked to complete a questionnaire about your background, your previous exposure to research and your feelings towards a research career. This study is voluntary. If you decide not to participate this will not impact your academic standing in any way. If you decide to take part, you will be asked to complete the survey by clicking on the link below. This survey is expected to take about 10 -15 minutes to complete, but there is no time limit and you can take as much time as you like. No background knowledge is required. We will ask for your consent for the collection and storage of data in accordance with the UK General Data Protection Regulation (GDPR) within the survey. For more information on GDPR please click on the following link: https://gdpr-info.eu/.
- **Do I have to participate?** Please note that your participation is voluntary. You may withdraw at any point during the questionnaire for any reason, before submitting your answers, by closing the browser. In cases of withdrawal from the study, no new data will be collected or linked to other data from that point on. If you do not want to answer some of the questions you do not have to, but you can still be in the study. All questions are optional. Your decision whether or not to be part of the study will not affect your academic standing or your access to university support services. If you have already submitted data and wish to withdraw from the study, please contact soham.bandyopadhyay@st-hildas.ox.ac.uk by 1st October 2021.
- **Who has approved this study?** This project has received ethics clearance through the University of Oxford's ethical approval process for research involving human participants, reference R73479/RE001.
- **How will my data be used?** Your answers will be completely anonymous, and we will take all reasonable measures to ensure that they remain confidential. Your data will be stored in a password-protected file and may be

used in academic publications. Your IP address will not be stored. If you provide us with your email address, we will delete that information at the end of the study. No answers will be linked to your email address. Research data – your anonymised answers – will be stored for a minimum of ten years after publication or public release.

- **Who will have access to my data?** Qualtrics is the data controller with respect to the personal data they hold about you and, as such, will determine how your personal data is used. Please see their privacy notice here: https://www.qualtrics.com/privacy-statement. Qualtrics will share any email address you provide and your anonymised answers with the University of Oxford, for the purposes of research. Researchers involved in the project will have access to this anonymised data. The University of Oxford is the data controller of university email addresses, please see their privacy notice here: https://compliance.admin.ox.ac.uk/student-privacy-policy. Responsible members of the University of Oxford and funders may be given access to data for monitoring and/or audit of the study to ensure we are complying with guidelines, or as otherwise required by law.
- **Are there any benefits to taking part?** Despite not have any immediate individual benefits by participating in this survey, you are given the opportunity to contribute to valuable and innovative research which could be used in the future by medical universities and the world. You may find this survey an opportunity to self-reflect. There will be the option to submit email address in order to be entered into a prize draw. At the conclusion of data collection, two random participants will be awarded £50 in Amazon vouchers, two further random participants will be awarded £25 in Amazon vouchers. This will be optional, as it requires you to provide personally identifying data (i.e. contact details). These will not be linked to the questionnaire answers given, and will only be used for contact regarding relevant rewards as above.
- **Will the research be published?** The findings of the study may be published in peer reviewed journals, presented at relevant conferences and meetings and a summary of the findings will be made available on social media**.**
- **Are there any possible risks involved with my participation?** Some of the questions that we ask may cause upset. If you experience any distress from participating in this study, you may stop the survey at any time or skip any upsetting questions. If your distress continues after leaving the survey, we have provided a list of supportive services nationwide that can be helpful and that you might consider contacting (appendix S5, to be linked here, and appear again at the close of the survey).
- **Who do I contact if I have a concern about the study or I wish to complain?** If you have a concern about any aspect of this project, please speak to the researcher soham.bandyopadhyay@st-hildas.ox.ac.uk who will do their best to answer your query. The researchers should acknowledge your concern within 10 working days and give you an indication of how they intend to deal with it. If you remain unhappy or wish to make a formal complaint, please contact the Chair of the Medical Sciences Inter-Divisional Research Ethics Committee: Email: ethics@medsci.ox.ac.uk; Address: Research Services, University of Oxford, Wellington Square, Oxford OX1 2JD OR The Chair will seek to resolve the matter in a reasonably expeditious manner.

For peer review only

- **How do I find out what was learned in this study?** This study is expected to be completed by approximately March 2022. If you would like a brief summary of the results, please write to us by email to request information
- **Who to contact for further details?** For any further questions or more information on the study, please contact us on the following email address: soham.bandyopadhyay@st-hildas.ox.ac.uk. Alternatively, you could contact principal investigator Dr Catherine Swales at catherine.swales@ndorms.ox.ac.uk.

Please note that you may only participate in this survey if you are 18 years of age or over.
☐ I certify that I am 18 years of age or over

If you have read the information above and agree to participate with the understanding that the data (including any personal data) you submit will be processed accordingly, please check the relevant box below to get started.
☐ Yes, I agree to take part

Tick this box if you would like to be considered for the prize draw, and are happy to be contacted via your email address for such
☐ Yes, I would like to be entered into the draw

Appendix S6

Many thanks for filling out the SMART questionnaire. We ask you to kindly give us a further few minutes of your time to answer questions regarding the questionnaire experience in order to improve it for future users.

Approximately how many minutes did it take you to complete the questionnaire? *Numerical*
In your opinion was the questionnaire *too long/ just right/ too short*?
Did you find the questions clear and easy to understand? *Yes/No*
Does the questionnaire omit any issues or factors you consider important to investigate? *Yes/No with white space*
Did you have any problems completing the questionnaire? *White space*
Is there anything else you would like us to know about the questionnaire, or the distribution of the questionnaire? *White space*

Appendix S7
We will utilise the test of parallel lines, and the output of our Multiple Correspondence Analysis to test initial statistical assumptions. We may relax the assumptions of the model by allowing partial proportional odds, or a generalised ordinal logit to ensure valid statistical analysis. We have used the directed acyclic graph approach to minimize bias introduction for causal inference from our predictive model, through selective covariate and predictor variable selection.

For peer review only

Our proposed initial model is: **Going to pursue academic career = (Ethnicity + Gender + Medical School + Free school meals + 1st Degree relatives in academia + Year of study + LGBTQ+) (**Equation 1).

Equation 1.

$$logit[P(\text{Going to pursue academic career} \leq j)] = \alpha_j + \beta_{Ethnicity}X_{Ethnicity} + \beta_{Gender}X_{Gender} + \beta_{Medical\ School}X_{Medical\ School} + \beta_{Free\ School\ Meals}X_{Free\ School\ Meals} + \beta_{Relatives\ in\ academia}X_{Relatives\ in\ academia} + \beta_{Year\ of\ study}X_{Year\ of\ study} + \beta_{LGBTQ+}X_{LGBTQ+}.$$

Where ‘Desire to pursue academic career’ represents answers to Question 20. (How much do you agree with the statement: “I wish to pursue an academic career or an academic training pathway .”). j represents the number of possible answers to Question 20 – 1. Ethnicity represents answers to Question 4. (Choose one option that best describes your ethnic group or background?) Gender represents answers to Question 5 (What gender do you identify as?). Medical School represents answers to Question 1a (Which medical school do you attend? (If currently intercalating at a separate university, please give the university from which you will receive your main degree)). Free School Meals represents answers to Question 7. (During School, at any point, were you eligible for free school meals?). Relatives in academia represents answers to Question 9. (How many of your first degree relatives are or have been in academia?). Year of study represents answers to Question 1b. (What year of medical school are you currently in?). LGBTQ+ represents answers to Question 6. (Do you identify as LGBTQ+?).

We will utilize likelihood ratios, Akaike information criterion, Schwarz criterion, -2 log likelihood, Pseudo-R^2 (Cox-Snell) to ascertain if this model has acceptable fit. If this full model does not, then we will optimise for a single model out of the set of possible nested models which will then be used as our subsequent model. We will utilise sampling weights identified in 1.5.

Appendix S8

Our proposed initial model is: **Research in future = (Ethnicity + Gender + Medical School + Free school meals + 1st Degree relatives in academia + Year of study + LGBTQ+) (**Equation 2).

Equation 2.

$$logit[P(\text{Desire to research in future} \leq j)] = \alpha_j + \beta_{Ethnicity}X_{Ethnicity} + \beta_{Gender}X_{Gender} + \beta_{Medical\ School}X_{Medical\ School} + \beta_{Free\ School\ Meals}X_{Free\ School\ Meals} + \beta_{Relatives\ in\ academia}X_{Relatives\ in\ academia} + \beta_{Year\ of\ study}X_{Year\ of\ study} + \beta_{LGBTQ+}X_{LGBTQ+}.$$

Where the above variables represent the same as in Appendix S7, apart from Desire to research in the future represents answers to Question 21.

Appendix S9

Going to pursue academic career = (Ethnicity + Gender + Medical School + Free school meals + Why did research + previous Degree + Year of study + LGBTQ+) (Equation 3).

Equation 3.

$$logit[P(\text{Going to pursue academic career} \le j)] = \alpha_j + \beta_{Ethnicity}X_{Ethnicity} + \beta_{Gender}X_{Gender} + \beta_{Medical\ School}X_{Medical\ School} + \beta_{Free\ School\ Meals}X_{Free\ School\ Meals} + \beta_{Why\ did\ research}X_{Why\ did\ research} + \beta_{Previous\ degree}X_{Previous\ degree} + \beta_{Year\ of\ study}X_{Year\ of\ study} + \beta_{LGBTQ+}X_{LGBTQ+}.$$

Going to pursue academic career = (Ethnicity + Gender + Medical School + Free school meals + Barriers to voluntary research + Year of study + LGBTQ+) (Equation 4).

Equation 4.

$$logit[P(\text{Going to pursue academic career} \le j)] = \alpha_j + \beta_{Ethnicity}X_{Ethnicity} + \beta_{Gender}X_{Gender} + \beta_{Medical\ School}X_{Medical\ School} + \beta_{Free\ School\ Meals}X_{Free\ School\ Meals} + \beta_{Barriers\ to\ voluntary\ research}X_{Barriers\ to\ voluntary\ research} + \beta_{Year\ of\ study}X_{Year\ of\ study} + \beta_{LGBTQ+}X_{LGBTQ+}.$$

Where all previously defined variables are as defined in Appendix 7 or Appendix 8. ‘Why did research’ represents answers to Question 17. (Why did/do you do research?). ‘Previous degree’ represents answers to Question 2. (Have you already completed an academic degree?). Barriers to voluntary research represents the set of common themes identified in each answer to Question 15. (If you have not yet been involved in voluntary research, what have been the barriers preventing this?).]

For peer review only
